# A review on microfluidic-assisted nanoparticle synthesis, and their applications using multiscale simulation methods

**DOI:** 10.1186/s11671-023-03792-x

**Published:** 2023-02-17

**Authors:** Abdulrahman Agha, Waqas Waheed, Ion Stiharu, Vahé Nerguizian, Ghulam Destgeer, Eiyad Abu-Nada, Anas Alazzam

**Affiliations:** 1 Department of Mechanical Engineering, Khalifa University, Abu Dhabi, UAE; 2 System on Chip Center, Khalifa University, Abu Dhabi, UAE; 3 Concordia University, Montreal, QC Canada; 4 École de Technologie Supérieure ÉTS, Montreal, QC Canada; 5 Department of Electrical Engineering, School of Computation, Information and Technology, Technical University of Munich, Munich, Germany

## Abstract

Recent years have witnessed an increased interest in the development of nanoparticles (NPs) owing to their potential use in a wide variety of biomedical applications, including drug delivery, imaging agents, gene therapy, and vaccines, where recently, lipid nanoparticle mRNA-based vaccines were developed to prevent SARS-CoV-2 causing COVID-19. NPs typically fall into two broad categories: organic and inorganic. Organic NPs mainly include lipid-based and polymer-based nanoparticles, such as liposomes, solid lipid nanoparticles, polymersomes, dendrimers, and polymer micelles. Gold and silver NPs, iron oxide NPs, quantum dots, and carbon and silica-based nanomaterials make up the bulk of the inorganic NPs. These NPs are prepared using a variety of top-down and bottom-up approaches. Microfluidics provide an attractive synthesis alternative and is advantageous compared to the conventional bulk methods. The microfluidic mixing-based production methods offer better control in achieving the desired size, morphology, shape, size distribution, and surface properties of the synthesized NPs. The technology also exhibits excellent process repeatability, fast handling, less sample usage, and yields greater encapsulation efficiencies. In this article, we provide a comprehensive review of the microfluidic-based passive and active mixing techniques for NP synthesis, and their latest developments. Additionally, a summary of microfluidic devices used for NP production is presented. Nonetheless, despite significant advancements in the experimental procedures, complete details of a nanoparticle-based system cannot be deduced from the experiments alone, and thus, multiscale computer simulations are utilized to perform systematic investigations. The work also details the most common multiscale simulation methods and their advancements in unveiling critical mechanisms involved in nanoparticle synthesis and the interaction of nanoparticles with other entities, especially in biomedical and therapeutic systems. Finally, an analysis is provided on the challenges in microfluidics related to nanoparticle synthesis and applications, and the future perspectives, such as large-scale NP synthesis, and hybrid formulations and devices.

**Graphical abstract:**

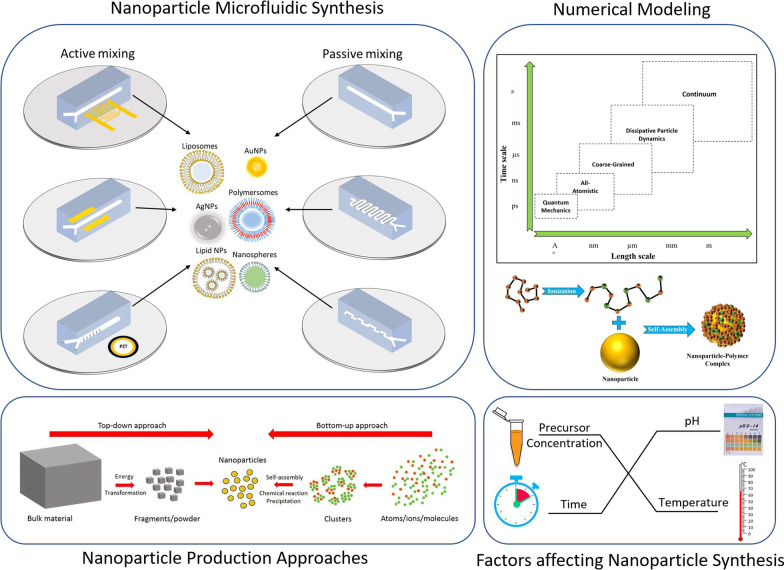

## Article highlights

- In this review article we have covered the state-of-the-art microfluidic methodologies for the synthesis of a broad range of nanoparticle (NPs) for potential applications in the fields of biomedicine and drug delivery.
- Apart from the experimental methodologies, this review also details the most common multiscale simulation methods used to unravel the critical mechanisms involved in nanoparticle synthesis and their interactions with other entities.
- A comprehensive summary of the microfluidic techniques, divided into passive and active micro-mixing methods, have been provided, while highlighting advantages and disadvantages of individual methods.
- We have discussed challenges related to NPs synthesis, their application in new fields, and future perspectives.

## Introduction

The utilization of nanoparticles (NPs) in the medical and pharmaceutical fields holds great potential for applications in drug discovery [1, 2], translational medicine [3, 4], clinical diagnosis [5, 6], and disease prevention [7–10]. NPs with their unique physicochemical properties exhibit distinct physical (e.g., electrical [11], and optical [12]), chemical [13], and biological properties (e.g., solubility, and toxicity [14]). As a drug carrier, NPs tend to enhance the stability and solubility of the encapsulated drug by protecting them from rapid metabolism and clearance while decreasing toxicity due to their controlled drug release and specific delivery [15]. For example, many newly discovered drugs are hydrophobic, making them difficult to be conventionally administered due to their low solubility and bioavailability [16]. However, when encapsulated in NPs, drug dissolution is improved due to high surface area-to-volume ratio offered by the NPs. Moreover, NPs can be engineered to escape clearance by the immune cells, and target specific sites in the body, e.g., micro-tumor environments [17]. This feature is of tremendous importance, especially to cancer patients, as conventional antitumor chemotherapeutics are non-specific, therefore, toxic to both the normal cells and cancerous cells [18]. In this regard, NPs can reduce the harmful side effects of cancer treatments and improve drug efficacy by increasing the drug circulation time and availability to the tumor [19]. As a result, there has been tremendous interest over the past decade to engineer NPs for targeted drug delivery [15, 20, 21]. Furthermore, due to the unique qualities of NPs, the field of nanotechnology is diverse with a wide range of applications in gene therapy [10], photocatalysis, catalytic reduction [22], electrochemical sensing [23, 24], energy storage, and environmental remediation [25]. For example, solid lipid nanoparticles (SLN) were used as gene carriers in the treatment of degenerative retinal disorders in rat models [26] and poly lactic-co-glycolic acid (PLGA) NPs were used in the delivery of locked nucleic acids for the treatment of cystic fibrosis [27]. Carbon nanomaterials are considered promising because of their excellent electrical, mechanical, and optical properties [28], where carbon NPs have been used in the removal of hazardous organic pollutants from seawater and groundwater [29], carbon nanotubes-graphene hybrid thin films were added to gold electrodes for the electrochemical detection of toxic arsenic (V) [30], and reduced graphene oxide-iron oxide NP nanocomposite electrodes were utilized for the detection of melatonin and dopamine [31]. Iron oxide NPs have attracted much attention due to its superparamagnetism and easy separation process (separation by a magnet), where they have been utilized as contrast agents in T_2_ magnetic resonance imaging [32], labeling of cells for tracking and monitoring of therapeutic delivery [33], drug delivery systems to deliver peptides, DNA, and chemotherapeutic drugs [34], and used as pigments to color construction materials [35] and in food coloring [36]. In addition, iron oxide NPs can be synthesized from precursors recycled from mill scale waste to prevent the possible contamination of soil and groundwater [37]. Moreover, silver chloride NPs have the ability to eliminate pollutants from water by the photocatalytic degradation of many dyes [38], functionalized gold and silver NPs were used for colorimetric sensing of glucose [39] and pesticides [40], gold NPs have shown to have anti-hyperglycemic effects in diabetic rat models [41] and were used in aqueous electrochemical capacitors for energy storage applications [42]. Copper oxide [22] and cadmium oxide [43] have shown to have antimicrobial activities against microorganisms.

There are numerous types of NPs depending on their chemical building blocks. These are broadly categorized as: organic (lipid and polymeric), inorganic (metals and metal oxides), and hybrid NPs. Each type is utilized in applications depending on the required characteristics. Organic NPs exhibit high biocompatibility and bioavailability [44, 45], whereas metal-based NPs can be synthesized with variable size, structure, and geometry [46]. Hybrid NPs are nanomaterials consisting of two or more distinct nanocomponents [47], where the formulated particles exhibit new or enhanced properties depending on the individual component. For example, combining magnetic NPs and PLGA NPs for simultaneous MRI imaging and drug delivery [48]. One of the earliest Food and Drug Administration (FDA) approved organic NP-based drug for cancer treatment, was Doxil® in 1995 [49], which consisted of lipid NPs (liposomes) loaded with doxorubicin and coated with poly(ethylene glycol) (PEG) to increase circulation time. Other FDA approved nanodrugs include the polymer-based Glatopa® [50] and iron oxide-based Feraheme® [51], which are used to treat multiple sclerosis and kidney disease, respectively. Recently, two liposome-based messenger RNA (mRNA) vaccines have been developed by Moderna, Inc. and BioNTech/Pfizer for the Severe Acute Respiratory Syndrome Coronavirus 2 (SARS‑CoV‑2) [9].

**Fig. 1:**
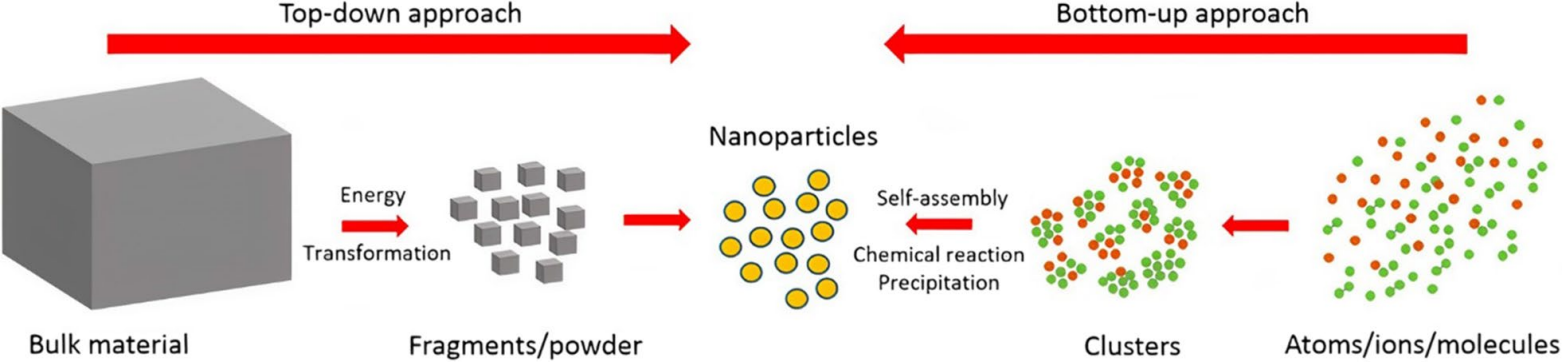
Schematic drawing of top-down and bottom-up approaches for nanoparticle production

Nanoparticle performance in vivo is governed by its physicochemical properties, and these include size, surface charge, morphology, and polydispersity index (PDI) [52]. Controlling these parameters is highly important for effective administration, correct dosage, and accumulation of NPs in target sites. For instance, liposomes in the range of 50–100 nm are required to evade the mononuclear phagocyte system (MPS) and accumulate into the leaky tumor vasculature due to the enhanced permeability and retention (EPR) effect. NPs in the size range of 20–100 nm can enter the spleen, bone marrow, and liver [53]. Thus, a wide size distribution (high PDI) could result in a significant portion of the administered nanodrugs being ineffective and cleared by the MPS. More specifically, the cellular uptake of NPs is carried out in a process called endocytosis [54] which involves three fundamentals steps (i) specific binding of NPs on cell surface, (ii) plasma membrane budding and pinching off to form endocytic vesicles, and (iii) transport to intracellular compartments. Depending on NP size, two types of endocytosis can occur, phagocytosis (i.e., cell eating) which uptakes NPs > 500 nm and is usually performed by macrophages, monocytes, and neutrophils, and pinocytosis (i.e., cell drinking) which engulfs fluids surrounding the cell and the suspended smaller NPs [55]. Surface charge is another important parameter influencing cellular uptake, NP stability, and interactions with the biological surroundings [56]. Where it is a major factor in the initial adsorption into the cell membrane prior to endocytosis [57]. For example, liposomes can either be cationic, anionic, or neutral depending on the charge of their constituent phospholipids, where cationic liposomes were found to be more effective in delivering drugs to the angiogenic blood vessels of solid tumors, neutral and anionic liposomes were used in drug delivery to the extravascular compartments of tumors [58]. In addition, NP stability in suspensions is determined by the zeta potential which depends on the surface charge, where values > + 30 mV and < − 30 mV indicate good stability against aggregation [59]. Moreover, the hydrophilicity and hydrophobicity of the administered drug carrier plays a role in its half-life and circulation time. Where hydrophobic NPs have a higher tendency to bind with plasma proteins (opsonins) in the bloodstream which are then eliminated by the MPS [60]. Thus, significant efforts are spent on the modification and functionalization of NP surfaces to escape the MPS and increase circulation time [61, 62]. One of the most common ways of functionalizing organic (lipid and polymer) NPs is by polyethylene glycol (PEG) coating (PEGylation) which is a nonionic hydrophilic polymer. Where after PEGylation, a hydrophilic protective layer is formed around the NP that reduces the adhesion of plasma proteins making them invisible to the MPS and increasing their circulation half-life by several times. Nanoparticles are conventionally synthesized by two approaches: top-down and bottom-up [63] (Fig. 1). Top-down methods involve mechanical work of macro-materials to produce nanoparticles followed by post processing steps such as extrusion and high-pressure homogenization to further decrease particle size. Because of its scalability, it is currently the preferred method in the industry [64]. However, this method is energy-demanding, requires expensive equipment, and produces particles with high PDI and batch-to-batch variation [65]. Examples of top-down methods include ball milling, thermal evaporation, and laser ablation [66]. On the other hand, bottom-up approaches rely on NP growth via precipitation from bulk mixing of precursors [67]. Since NP formation generally occurs in an environment with a millimeter or centimeter scale, local fluctuations in precursors' concentrations can develop that result in particle size heterogeneity and variation [68]. In comparison with top-down approaches, bottom-up techniques require less energy, space, and material to produce particles with better properties, thus making it suitable for research-setting synthesis at low cost [69, 70]. Bottom-up methods include co-precipitation and chemical vapor deposition [66, 71]. Detailed reviews of conventional (top-down and bottom-up) NP synthesis methods were presented in references [33, 38]. Overall, conventional methods lack precise control over experimental parameters, producing NP with a wide size distribution and batch-to-batch variation in physicochemical properties [72]. Other limitations include: the requirement of additional chemical and physical processes (freeze thaw, high pressure homogenization and extrusion), waste of resources, insufficient macro-mixing, and potential contamination. These drawbacks associated with conventional methods impede the translation of NP drugs from laboratory to clinical use [73]. Thus, it is a crucial matter to investigate and develop innovative techniques to address these challenges and synthesize NPs with high reliability and control.

An alternative approach to conventional methods is miniaturizing the synthesis process by utilizing microfluidic channels. Microchannels offer the ability to overcome the limitations of top-down approaches and bulk mixing by their micro-scale dimensions and mixing, precise control of flow parameters, particle size tunability, and reproducibility [74]. Similar to bulk mixing of bottom-up approaches, microfluidic NPs are affected by various factors including temperature, precursor concentration, time, and pH. However, due to the continuous flow operation of microfluidics, additional factors such as total flow rate (TFR), flow rate ratio (FRR) and residence time also influence these parameters and the physicochemical properties of NPs. Where TFR is the sum of flow rates entering a microchannel, and FRR is the ratio of aqueous flow rate to NP precursor flow rate. These flow parameters are closely related to the concentration and time in microfluidic synthesis. Of these factors, FRR is more influential on NP size and PDI. Where, as FRR increases (higher ratio of aqueous flow rate to NP precursor) shear stress between the streams increase, and the width of precursor stream is decreased (lower concentration of NP precursor). This results in a shorter mixing time and length, and smaller NP formation. Moreover, pH gradients in aqueous solvents can be used in liposome drug loading [75] and produce smaller lipid nanoparticles at more acidic conditions (6 vs 7.4) [76]. Elevated temperatures can be used to speed up chemical reaction, facilitate in fluid mixing, and synthesize smaller NPs. For example, when using distearoylphosphatidylcholine (DSPC) for liposome synthesis, temperatures above the transition temperature tend to decrease liposome size [77].

Microfluidic devices can be fabricated from a variety of materials, including glass [78], silicon [79], polydimethylsiloxane (PDMS) [80], lithium niobate (LiNbO_3_) [81], and cyclic olefin copolymer (COC) [82, 83]. In addition, depending on the material, different fabrication techniques can be employed, for example, soft lithography for PDMS, and etching for glass and silicon [84], and hot embossing for COC [85]. Jahn et al. [86] in 2004 were the first to produce liposomes in a microfluidic hydrodynamic focusing (MHF) channel. Liposome size was controlled by the tuning of solvent flow rates and concentrations. Following this breakthrough, microfluidic-based liposome synthesis gained much attention from the engineering, medical, and pharmaceutical fields [76, 87–89]. However, since the flow in microfluidics is characterized by a low Reynolds number, mass transfer occurs by diffusion between the laminar streams, therefore, MHF devices are limited by diffusion to mix NP precursors, which results in a long mixing time and low mixing efficiency [90]. Thus, the next step in the evolution of the microfluidic NP synthesis was to induce mixing inside microchannels to overcome the laminar flow nature of microfluidics. Mixing is achieved in two ways in microchannels: passive and active mixing. Passive mixing relies on inducing flow disturbances by designing unique geometrical structures in the microchannel, such as staggered herringbone [91], serpentine [92], and tesla [93] structures. Passive microchannels have a simple setup and fabrication and can operate at high flowrates. However, tunability is only achieved by changing the flow rates. Whereas active mixing depends on external physical fields such as acoustic [94] and electric [95], to generate body or surface forces on the fluids and induce mixing. With this approach, tunability can be achieved by altering the flow rate and changing the external field parameters. However, fabrication is generally more complicated, and the devices are operated at relatively low flowrates.

In this article, a comprehensive review of advances in microfluidic-synthesized NPs is presented. Several review articles related to NP formulation and applications were published in the past [73, 96–99] However, given the rapid advancements in the synthesis and application potentials of nanoparticles, it is imperative to update the knowledge and keep up with all the newest developments and trends in this research area. Furthermore, the recent review articles [73, 96–99] have given less emphasis on active microfluidic NP synthesis and more attention on the progress in passive microfluidic methods to synthesize NPs. In contrast, the novelty of the current work lies in the detailed insight into the fundamental driving mechanisms and progress in active and passive microfluidic mixing techniques for NP production. In addition, a significant portion of the current work is dedicated to understanding the basics of multiscale computational methods and their advancements in synthesizing nanoparticles and understanding the roles of nanoparticles at molecular levels in different physical phenomena. The computational methods hold great importance in nanoparticle research because they provide an alternative to the real experiments and allow the researchers to perform “computer experiments” where mimicking physical conditions is difficult in the real-world experiments. The remaining sections of this article are organized as follows: “Types of NPs” Section presents various types of nanoparticles and their advantages and disadvantages. “Micromixing for NP production” Section provides a detailed description of the passive and active mixing methods to synthesize NPs. “Computational Approaches to study NPs” and “Molecular Simulations of Nanoparticles” Sections discuss the available computational approaches and progress to simulate systems involving nanoparticles. Finally, “Conclusions, outlook and future aspects” section provides the conclusion and future perspective in the field of NPs and microfluidics.

## Types of NPs

In this section, a brief introduction is presented on several types of NPs including lipid NPs, polymer-based NPs, and inorganic NPs.

### Lipid-based NPs

Early work on lipid-based nanoparticles began in the 1960s by Alec Bangham [100]. Initially, they were considered a model to study cell membrane functions due to their structural similarity. The shift to lipid drug delivery system applications started later in the 1970s [101]. Lipid NPs can be further classified into various subsets depending on their structure and formulation [102]. However, they are generally spherical vesicles with at least a single lipid bilayer and an internal compartment. As a carrier, lipid NPs have the advantages of self-assembly, biodegradability, biocompatibility, bioavailability, simple synthesis, and surface functionalization [15]. As a result, liposomes constitute the most NP-based drugs approved by the FDA [103].

Liposomes are one of the most studied drug delivery systems due to their biocompatibility, bioavailability, and ability to carry hydrophilic and lipophilic drugs simultaneously [19, 88]. They are spherical vesicles composed of phospholipids with one (unilamellar) or multilayer (multilamellar) membrane separating the inner aqueous core from the external environment. Its synthesis starts with a polar solvent such as water and a nonpolar organic solution. The nonpolar solution is comprised of phospholipids (e.g., phosphatidylcholine (PC), 1,2-distearoyl-sn-glycero-3-phosphocholine (DSPC), and 1,2-dioleoyl-sn-glycero-3-phosphatidylethanolamine (DOPE)) and an organic solvent miscible in both water and lipids such as ethanol or isopropanol [104]. As the polar and nonpolar solvents mix, phospholipids close upon themselves, forming liposomes in a process called self-assembly [105]. Depending on the type of application and specific requirements for liposomes, different phospholipids can be incorporated into the process. For example, DOPE is added to prepare pH-sensitive liposomes and DSPC enhances the drug amount and release rate [106]. In addition, cholesterol modifies the fluidity/elasticity of liposomes [107]. However, liposomes show low drug encapsulation efficiency and leakage [108]. Table 1 shows the advantages and disadvantages of lipid-based NPs along with other types of NPs.

**Table 1:** Advantages and disadvantages of different nanoparticles as delivery systems

	Nanoparticle	Advantages	Disadvantages
Lipid based	Liposomes SLN Emulsions	Formulation simplicity High bioavailability Payload flexibility Biodegradable Biocompatible	Low encapsulation efficiency Poor stability Leakage May trigger immune response
Polymer based	Nanocrystals Nanospheres Polymersomes Dendrimers	Biodegradable Payload flexibility Precise control of characteristics Easy surface modification	Possibility of aggregation Toxicity Low cell affinity
Inorganic based	AuNP AgNP Iron oxide Quantum dots	Unique electrical, optical, and magnetic properties Variable size, structure, and geometry Easy functionalization	Poor solubility Toxicity

Another class of lipid NPs are solid lipid nanoparticles (SLN) developed in the 1990s to overcome the low efficiency of drug encapsulation in liposomes [108, 109]. They can be synthesized from various lipids, including mono-di- or triglycerides, glyceride mixtures, and lipid acids that remain in a solid state in vivo and at room temperature [19]. Generally, SLNs are modified with polyethylene glycol (PEG) to improve stability and circulation [110]. In addition, they are distinguished from liposomes in that they possess a solid matrix core to encapsulate drugs [15]. SLNs exhibit significant advantages, including physical stability, site-specific targeting, and controlled release. However, they are still limited by drug loading and leakage.

### Polymer-based NPs

**Fig. 2:**
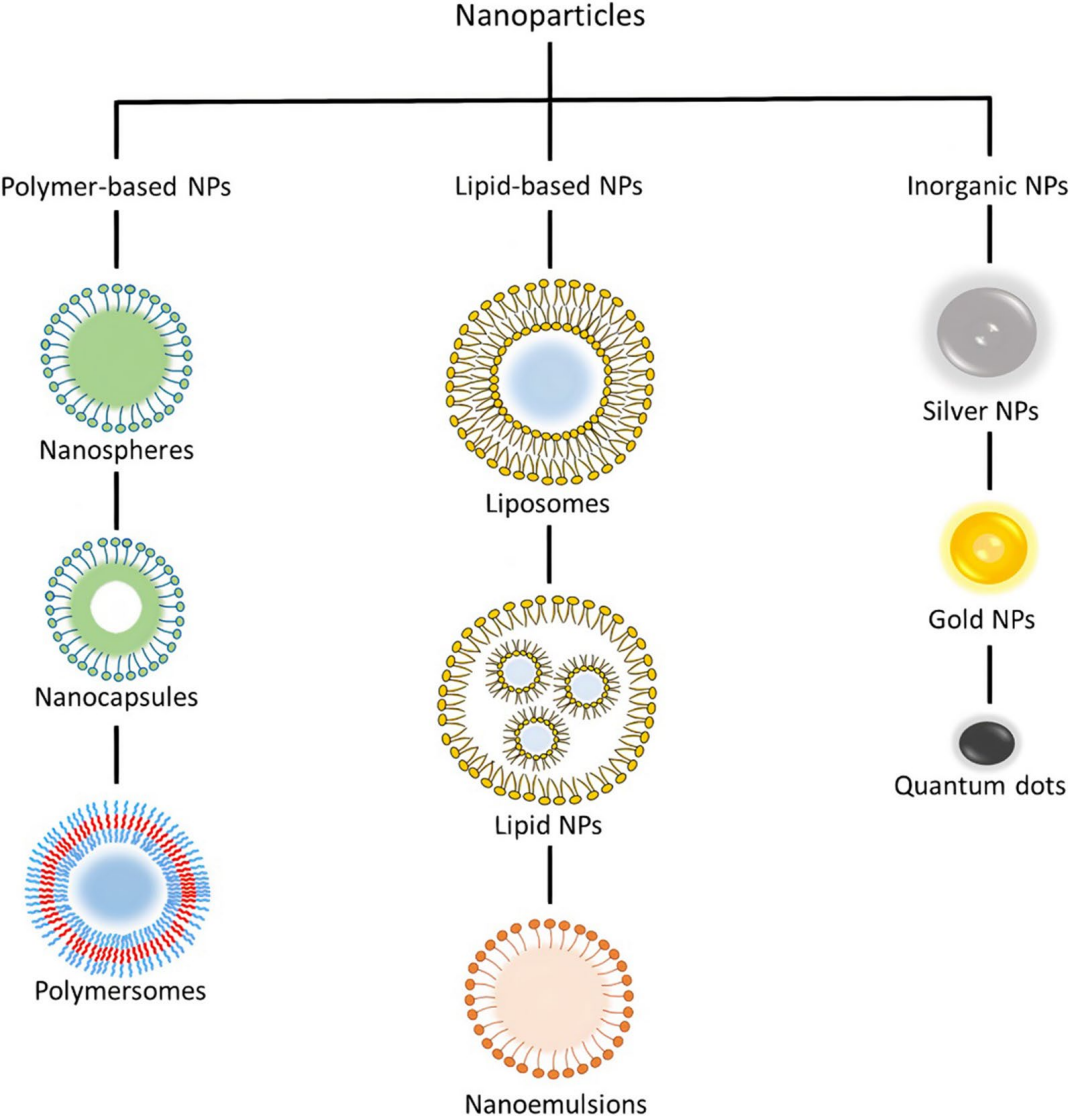
Different classes of nanoparticles

Polymer-based NPs have been investigated for applications in drug delivery and contrast imaging since the 1970s [111, 112]. They were developed because of their biocompatibility, biodegradability, stability and can be prepared to enable precise control of NP characteristics and action duration [113]. They can be synthesized from synthetic or natural polymers. Synthetic polymers include poly(lactide-co-glycolide) (PLGA), poly(lactic acid) (PLA), polyanhydrides, and polycaprolactone (PCL), whereas natural polymers include chitosan, gelatin, hyaluronan, and alginate [113, 114]. Figure 2 shows the different types of polymer-based NPs along with other classes of NPs. In addition, they can be synthesized using various methods such as nanoprecipitation [115], emulsification [116], and microfluidics [117]. Depending on the material and technique used, different final NP products are obtained with a variety of structures and characteristics [15]. A subset of polymer-based NPs is polymeric NPs, which are solid colloidal systems where the therapeutic material is either encapsulated, dissolved, or absorbed into the polymer matrix. Depending on the formation process, these NPs can be classified as nanospheres, which are matrix systems where the drug is dispersed throughout the NP [118], and nanocapsules, which are vesicular systems where the drug is entrapped in an oily liquid surrounded by a single polymer layer. Nanospheres and nanocapsules are typically synthesized from PLGA, PLA, PCL, and chitosan [118]. On the other hand, if the core of the vesicular system is aqueous and surrounded by amphiphilic block copolymers, the NP is referred to as polymersomes, which are analogous to liposomes and are formed by self-assembly [119].

### Inorganic NPs

Inorganic NPs include materials such as gold (AuNP), silver (AgNP), metallic oxides, and semiconductors. They are used as drug carriers and imaging contrast agents because of their unique optical, magnetic, and electrical properties [98]. In addition, these NPs can be engineered into a variety of structures and geometries. AuNPs are among the most studied inorganic NPs and can be synthesized in various forms, such as nanorods, nanocubes, nanoshells, nanospheres, and nanostars [120, 121]. Magnetic NPs are usually composed of iron oxide or ferrites such as magnetite (Fe_3_O_4_), metallic NPs (iron and cobalt), or alloy NPs (cobalt–platinum alloys) [122]. Where iron oxide NPs make up the majority of inorganic approved nanomedicines by the FDA [123]. Quantum dots are a class of NP typically made of semiconductors such as silicon, germanium [123], and cadmium [124].

### Hybrid NPs

Hybrid NPs can be defined as nanomaterials consisting of two or more distinct types of nanocomponents [47]. Where different types of materials are combined to enhance certain properties, synthesize hybrid NPs with new unique properties, or to overcome limitations of individual NPs [125]. Hybrid NPs can be broadly categorized into three groups: organic/organic NPs, organic/inorganic NPs, and inorganic/inorganic NPs. Each of these combinations can be customized in terms of constituent material and structure by tuning their ratios to result in the optimal hybrid NP for the selected application [126]. Organic/organic NPs are hybrid NPs consisting of various types of lipids and polymers that combine the biomimetic characteristics of lipid NPs and the structural properties and stability of polymer NPs to improve the nano-carrier system [127]. Examples of lipid-polymer hybrid NPs include polymer core–lipid shell hybrid NPs, polymer-caged nanobins, and monolithic polymeric lipid hybrid NPs/mixed polymer–lipid hybrid NPs [125]. Organic/inorganic hybrid NPs combine organic materials such as lipid or polymer NPs with metal, metal oxide, or semiconductor materials to take advantage of their unique electrical, optical, and magnetic properties. For example, magnetoliposomes [128] consist of a magnetite NPs encapsulated in liposomes for applications in molecule separation and targeted drug delivery. Other examples include zeolitic imidazolate framework NPs (metal organic framework (MOF)) [129], and gold (core)–polystyrene (shell) NPs [130], in addition to numerous organic/inorganic core–shell combinations [131]. Similarly, inorganic/inorganic hybrid NPs can be made from a combination of metals, metal oxides, and semiconductors, such examples include gold NPs (unique optical properties) coated with a silica nanometric layer (high stability), NP–quantum dot NPs, and core–shell bimetallic NPs [47].

## Micromixing for NP production

Efficient mixing is crucial for the synthesis of NPs, where the interaction of fluids causes the generation of NPs [132]. Mixing time and uniformity dictate the size and size distribution of NPs [133], such that a shorter mixing time results in smaller particles and a lower PDI. In microfluidics and bottom-up methods, size and PDI depend on the interplay between mixing time (*t*_mix_) and precipitation time (*t*_precipitation_) [134]. When the mixing time is less than the precipitation time (*t*_mix_ < *t*_precipitation_), smaller NPs are produced with lower PDI as solvents and antisolvents mix in a timeframe less than the time required for precipitation to occur, and vice versa (*t*_mix_ > *t*_precipitation_). In bottom-up methods, NP synthesis occurs in a millimeter or centimeter scale environment, leading to local fluctuations of concentration that result in large particle size and size distribution (*t*_mix_ > *t*_precipitation_) [68]. In contrast, mixing in microfluidics occurs at a micrometer scale, where efficient mixing can result in *t*_mix_ < *t*_precipitation_. However, microchannels are generally known for their laminar flow nature [135], and diffusion-based mixing (straight microchannel), characterized by a low Reynolds number (Re ~ 1):

1 $${\text{Re}} = \frac{{\rho {\text{UD}}_{h} }}{\eta }$$

where *ρ* is the fluid density, *U* is the fluid velocity, *D*_*h*_ is the hydrodynamic diameter of the channel, and *η* is the dynamic viscosity of the fluid. For example, water with *ρ* ~ 1000 kg/m^3^ and, *η* ~ 0.001 Pa s, in a microchannel with a *D*_*h*_ of 100 µm, the Re of the flow approaches 1 for a mean flow velocity of 0.01 m/s, which is commonly achieved in microfluidic operations [136].

Given the intrinsic properties of microfluidic channels (channel size, fluid velocity, density, and viscosity), inducing vortices and chaotic mixing by transitioning to turbulent flow (Re > 2000) is out of the question. Thus, passive and active mixing strategies are implemented to achieve efficient mixing and overcoming diffusion. Another important dimensionless number is the Peclet number, which characterizes the type of mixing in microchannels, given in Eq. 2, where it compares advection transport to diffusion transport. Chaotic mixing enhances advection transport and increases the Peclet number [137]. It can also be interpreted as the ratio of diffusion time to advection time. Lower advection time (high mixing rate) leads to a higher Peclet number:

2 $${\text{Pe}} = \frac{{{\text{UD}}_{h} }}{D} = \frac{{t_{D} }}{{t_{A} }}$$

where *U* is the fluid velocity, *D*_*h*_ is the hydrodynamic diameter of the channel, *D* is the diffusivity, t_D_ is the diffusion time, and t_A_ is the advection time, where the diffusion length *L*_*D*,_ *D*, and *t*_*D*_ are related by $L_{D} = \sqrt {Dt_{D} }$. A molecule with a diffusivity *D* of 10^–10 ^ m^2^/s will take approximately 100 s to diffuse across a 100 µm channel, a larger particle with 10^–11^ m^2^/s diffusivity will diffuse in 1000 s across the same width.

This section introduces the fundamentals of mixing in microchannels, followed by a comprehensive review of the literature concerning NP synthesis in microchannels along with the factors affecting their production such as concentration, temperature, flow rate (flow rate ratio and total flow rate), and time.

### Passive micromixing

Passive micromixers are microfluidic devices that use geometrical features and embedded microstructures to induce chaotic advection or fluid interruption for the purpose of fluid mixing.

### Background on passive mixing methods

This section is mainly concerned with the mixing techniques employed in the microfluidic devices without any involvement of the external fields. The process of mixing in microfluidics devices is a crucial step for preparing NPs because mixing a solvent with another solvent (or an anti-solvent) initiates the formation of NPs. The time required for mixing is directly linked to the diameter and size distribution (monodispersity) of the resulting NPs [105]. Small-sized NPs with excellent monodispersity are created by faster mixing processes in which the mixing time is less than the nucleation period of the nanoparticles. In contrast, slow mixing (where the mixing time exceeds the time required for NP nucleation) results in larger NPs with a broad range of sizes.

**Fig. 3:**
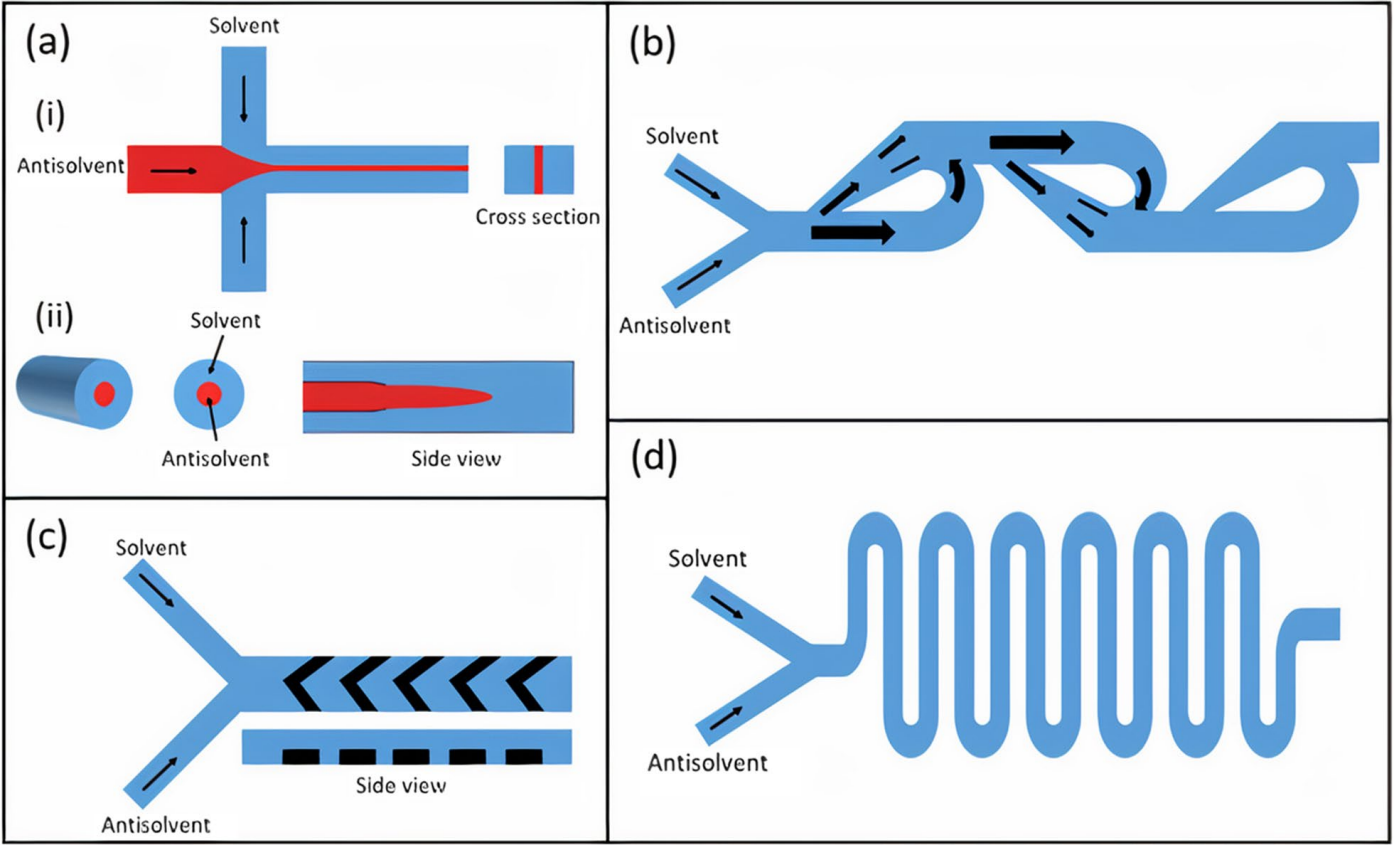
Passive mixing methods. **a** (i) 2D MHF, (ii) 3D MHF. **b** Microfluidic Tesla mixer. **c** Staggered herringbone micromixer. **d** Serpentine micromixer

Among the passive microfluidic mixing techniques, the simplest one is based on microfluidic hydrodynamic focusing (MHF). MHF employs multiple fluids flowing in parallel in the microchannel in the laminar regime (Re ~ 1) [135]. A central hydrodynamically focused fluid stream at a lower flow rate co-flows with outer sheath streams at relatively high flow rates. As a result, the diffusion length (viz. width of the inner stream) is reduced, which reduces the mixing time dramatically and facilitates faster mixing [138]. The decreased mixing time may also enhance the quality of the nanoparticles. The MHF is simple in operation since the most crucial feature for NP synthesis is the flow behavior of the participating solvents, which can be accurately manipulated by tuning their flow rates. Other controlling factors are the microchannel geometry and the choice of material. Thus, the quality of the synthesized NPs can be easily controlled using this technique. The MHF platforms can be classified into two types [139]: (i) planar or 2-D MHF platforms, and (ii) coaxial based or 3-D MHF devices. In a 2-D MHF platform, the central fluid stream is focused in a single plane (Fig. 3ai), whereas, in a 3-D MHF platform, the outer sheath flow focuses the central solvent in both horizontal and vertical planes (Fig. 3aii). Ideally, a 3-D MHF platform is desired because it results in more uniform velocities in both planes. Nevertheless, the 2-D MHF platforms are ubiquitous owing to their ease of manufacturing and integration.

In general, MHF platforms have an easier fabrication process due to their simple, straight design compared to the other mixing platforms discussed below. Moreover, the technique is easier to simulate since it involves the surface tension force at the interface between the two liquid solvents. Hence, the outputs can easily be predicted before even manufacturing the prototypes to perform actual experiments. Furthermore, high flow rates may be accommodated in MHF, allowing for high-throughput applications [140, 141].

The second class of passive microfluidic mixing platforms involve the split-and-recombine (SAR) method of the fluid streams. In the SAR method (also termed as co-lamination), the microchannel splits in two branches forcing the fluids to follow in dissimilar path lengths, followed by the merger of channel branches which recombines the fluid streams [142]. Repeating the process multiple times induces rapid chaotic mixing. Chaotic mixing is a fast mixing process that relies on chaotic advection, which may be achieved by stretching and folding a fluid volume repeatedly [143].

Similar to MHF, SAR-based mixing can be achieved in-plane or out-of-plane. The in-plane SAR mixers are simpler to fabricate and thus more common. The two most efficient SAR-based in-plane mixers are: (i) the bifurcating mixer, and (ii) the Tesla mixer (Fig. 3b). The bifurcating mixer comprises a series of circular toroids that subjects the fluids under centrifugal forces to achieve a fast mixing process [144]. The second type of in-plane mixer, called the Tesla mixer, employs the Coandă effect, which is the propensity of a fluid initially flowing in a straight direction to follow and remain attached to a convex-shaped curved surface instead of flowing in the original direction [145]. By exploiting this effect and introducing alternate convex curves on the opposite sidewalls of the channel, the Tesla mixer achieves chaotic mixing efficiently.

Chaotic mixing can also be achieved in the microchannel by embedding microstructures, such as ridges, on the floor of the microchannel. The ridges are placed at an oblique angle at the bottom of the channel [146] and can be easily incorporated into the microchannel using standard lithography and soft-lithography techniques. These oblique ridges induce an anisotropic resistance to the fluid flow, which generates vortices in the flow, and enhanced chaotic mixing is obtained. Another architecture is to use the ridges that are in the shape of staggered herringbone [147]. In the staggered herringbone configuration (Fig. 3c), the ensuing circulating fluxes continually stretch and fold the fluid volume, thus enhancing the concentration gradient substantially.

Numerous microchannel architectures also aim to employ fluid inertial effects to perform passive mixing. As is known, the inertial effects are dominant relative to the viscous effects if Re > 1. Thus, the fluid is introduced into the microchannel at higher flow rates and if it encounters a curvature in the microchannel geometry, vortices are formed. These micro-vortices are formed because of the mismatch between the velocities of different portions of the fluid. Incorporating sudden expansions in the microchannel is also an effective way of generating micro-vortices in the microchannel [148]. This expansion can be planar (2-D) obtained by increasing the width of the microchannel, or 3-D, which is achieved by arranging tubes of different diameters in a coaxial arrangement. Another effective method that is used to obtain inertial-based mixing is based on using Dean flow, a secondary cross-sectional flow field obtained by introducing curvature in microchannels such as the serpentine micromixer (Fig. 3d) [149]. The Dean flow is characterized by the existence of two counter-rotating vortices that are perpendicular to the flow direction and are positioned above and below the channel's plane of symmetry.

#### Organic NP synthesis

By utilizing the aforementioned passive mixing mechanisms, numerous types of NPs have been fabricated in a controlled manner (Table 2).

**Table 2:** Microfluidic synthesis of nanoparticles by passive mixing methods

Nanoparticle type	Mixing mechanism/microchannel	Size (nm)	PDI	Organic/precursor concentration	FRR	TFR	ME	MT	Year	References
Liposomes	Concentric laminar flow/PDMS mixer	120	–	1 mM	4	20.4 µL/min	–	–	2012	[150]
Liposomes	Semicircular contraction–expansion array (Dean flow)/PDMS/glass microchannel	50	–	10 mM	9	18 mL/h	90%	–	2013	[151]
Liposomes	Periodic disturbance mixer (Dean flow)/PDMS/glass microchannel	30	0.22	5 mM	3	18 mL/h	> 90%	< 120 ms	2021	[152]
Liposomes	Serpentine microchannel with baffles (contraction–expansion regions)/PDMS/glass microchannel	118	0.2	25 mM	5	11.8 mL/min	–	–	2013	[153]
Liposomes	Staggered herringbone mixer/PDMS/glass microchannel	30	0.1	5 mg/mL	10	500 µL/min	80%	< 300 ms	2017	[105]
Lipid nanoparticle	2D-baffle PDMS mixer device (invasive lipid NP production device/PDMS/glass microchannel	20	0.1	10 mg/mL	20	500 µL/min	90%	6 ms	2018	[154]
Liposomes	Double flow focusing/PDMS/glass microchannel	150	0.23	5 mM	10	65 mL/h	–	–	2021	[141]
Lipid nanoparticle	Segmented-Axis symmetric glass microchannel	70	0.15	5 mg/mL	10	130 μL/min	–	–	2019	[157]
Liposomes	Periodic disturbance mixer (Dean flow)/PDMS/glass microchannel	90	0.18	10 mM	8.56	18 mL/h	–	–	2020	[164]
Liposomes	Serpentine/PMMA polymer microchannel	188	0.2	1.5 mg/mL	5	5 mL/min	–	–	2020	[156]
Liposomes	Half-moon design/PLA polymer microchannel	193	0.22	1 mg/mL	1	3 mL/min	–	–	2021	[165]
PLGA	3D origami ship spiral/arc design/PDMS-PDMS microchannel	< 100	0.06	2% wt	17	2.5 mL/h	–	16 ms	2013	[166]
PLGA	Slit interdigital micromixer	211	–	10 mg/mL	1	62.8 mL/min	–	10 ms	2016	[167]
PLGA	Spiral mixer (Dean flow)/PDMS/glass microchannel	69.3	0.2	5 mg/mL	5	132 mL/min	–	< 90 ms	2020	[169]
PLGA	Staggered herringbone mixer (NanoAssemblr™)/COC microchannel	120	0.12	10 mg/mL	3	6 mL/min	–	–	2018	[170]
PLCL	Serpentine design/Asia 320 microfluidic system	30	< 0.12	0.25 mg/mL	5	2 mL/min	–	–	2018	[191]
PCL	Concentric glass capillaries microchannel	200	–	1 mg/mL	10	8.3 mL/h	–	–	2015	[140]
PCL-b-PEO	Segmented flow-based mixer (Taylor flow)/PDMS/glass microchannel	32	–	0.66% wt	1	100 µL/min	–	< 1 s	2016	[168]
AuNPs	Spiral segmented flow-based mixer (Taylor flow)/silicon/glass microchannel	3.8 ± 0.3	–	1 mM	10	300 µL/min	–	–	2012	[171]
AuNPs	Serpentine design/PDMS/glass micorchannel	4.38 ± 0.53	–	10 mM	9:14	2.3 mL/h	–	–	2010	[172]
AuNPs	Integrated micromixer-microreactor-microfluidic platform/PDMS/PDMS microchannel	27 ± 3.5	–	0.02% wt	1	10 µL/min	83%	–	2015	[192]
AuNPs	Serpentine design/PMMA micorchannel	4.5	–	10 M	5:3	0.2 mL/min	–	–	2019	[177]
AgNPs	Double layer Y-shaped split and recombination micromixer/PMMA microchannel	30.5 ± 4.82	–	1 mM	1.5	810 μL/min	–	–	2020	[174]
AgNPs	T-mixer	4.7 ± 0.6	–	0.5 mM	1	1.9 mL/min	–	–	2015	[178]
AgNPs	Corning Advanced-Flow Reactor (AFR) curved split and recombination/glass microchannel	4.6 ± 1.8	–	1.58 mM	2	9 mL/min	60%	–	2021	[175]
Silica	Gear-shaped serpentine micromixer/PDMS/glass microchannel	370.3	–	0.1 M	–	1 mL/h	90%	–	2020	[176]
Lipid-PLGA	Tesla structure micromixer/PDMS/glass microchannel	40	–	1 mg/mL (PLGA)	10	60 μL/min	–	10 ms	2010	[181]
Lipid-QD	Tesla structure micromixer/PDMS/glass microchannel	60	–	0.5 mg/mL (QD)	–	55 μL/min	–	10 ms	2010	[181]
Lipid-PLGA	Spiral micromixer/PDMS/glass microchannel	62.5 ± 1.18	0.173	2.94 mg/mL (lipid), 10 mg/mL (PLGA)	80	246 mL/h	–	–	2015	[182]
Lipid-PLGA	Herringbone patterned multi-inlet vortex mixer	60	0.12	1 mg/mL (PLGA)	–	12 mL/min	> 99%	–	2019	[183]
Au-PLGA	Two consecutive slit-interdigital micromixers	192 ± 58	–	1.25 mg/mL of (PLGA), 0.24 mg/mL (gold)	–	54 mL/h	–	–	2017	[184]
Au-Lipid	Asia MF 320 system flow focusing laminar mixing	130	0.2	4.5 mg/mL(lipid), 2.5 mg/mL (gold)	10	220 μL/min	–	–	2019	[187]

*PDI* polydispersity index, *FRR* flow rate ratio, *TFR* total flow rate, *ME* mixing efficiency, *MT* mixing time

##### Lipid NPs

Kennedy et al. [150] described the liposomal self-assembly in a laminar flow PDMS mixer. The 3-inlet device has a square cross section and creates a cylindrical organic phase core surrounded by the aqueous phase to increase the interface contact area and prevent lipid deposition on channel surfaces. The organic phase was comprised of 1,2-dipalmitoyl-sn-glycero-3-phosphocholine (DPPC), 1,2-distearoyl-sn-glycero-3-phosphoethanolamine-N-[amino(polyethyleneglycol)-2000] (DSPE-PEG), and cholesterol (10:1:10) dissolved in ethanol. Liposomes with an average size of 100–200 nm were produced with a flow rate ratio (FRR) ranging from 5 to 20, and a total flow rate (TFR) of 20.4 μL/min. Lee et al. [151], reported on the synthesis of liposomes in a PDMS/glass semicircular contraction–expansion array (CEA) microchannel. The reported micromixer is based on Dean vortices where the geometrical features were only designed on one side of the channel. Dimyristoylphosphatidylcholine (DMPC) and cholesterol (1:1, 10 mM concentration) were dissolved in isopropanol as the organic phase. At a TFR of 18 mL/h and 9 FRR, the CEA device produced 50 nm NPs in comparison to 200 nm particles produced from a MHF channel. In addition, the highest mixing efficiency (90%) was achieved at a TFR ranging from 12 to 15 mL/h.

**Fig. 4:**
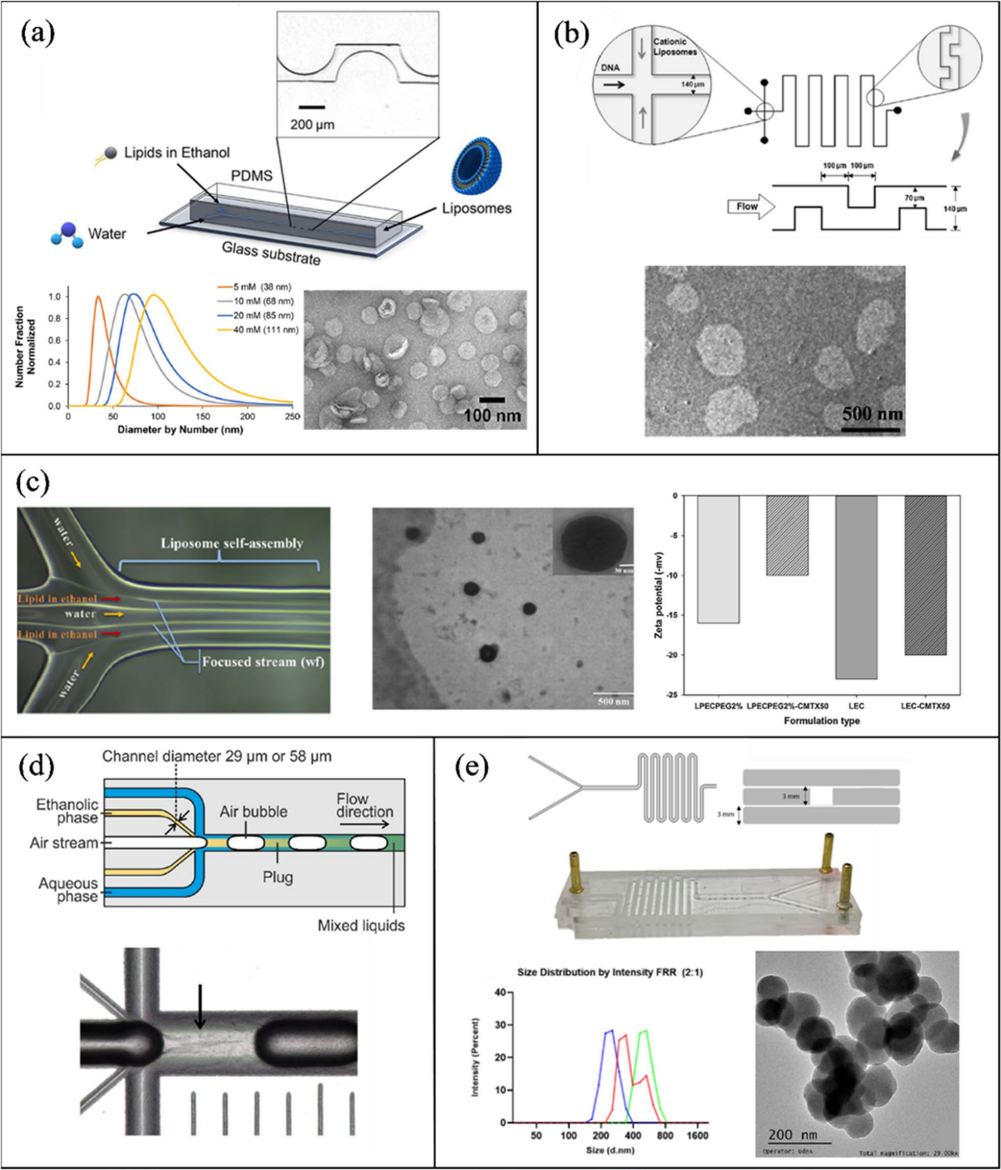
Passive microfluidic mixers for lipid-based NP synthesis. **a** Liposomes fabricated using periodic disturbance mixer. Reprinted with permission from [152]. Copyright 2021 American Chemical Society. **b** Serpentine microchannel with baffles for the synthesis of DNA/cationic liposomes complexes. Reproduced with permission from [153]. Copyrights © 2013 Elsevier. **c** MTX loaded liposomes in a double flow focusing microchannel. Reprinted with permission from [141]. Copyright 2019 American Chemical Society **d** LNP fabrication in a segmented flow glass microchannel. Reproduced with permission from [155]. Copyrights © 2020 Elsevier. **e** PMMA serpentine platform for the synthesis of LNP (open access) [156]

In a different work, López et al. [152] utilized a PDMS/glass CEA micromixer, termed “periodic disturbance mixer” (PDM) to conduct a parametric study on factors affecting liposome size and PDI. At a constant FRR of 8.56 and temperature of 70 °C, increasing the TFR from 5 to 20 mL/h decreased liposome (DMPC, cholesterol, dicetyl phosphate (DHP), 5:4:1) size from 50 to 30 nm with a constant PDI of 0.22 (measured by dynamic light scattering (DLS)). On the other hand, varying the FRR from 1 to 3 at a constant TFR of 18 mL/h reduced the size from 120 to 35 nm. This makes the PDM valuable in comparison to other devices, as it produces small NPs with good PDI at a low FRR of 3. Morphological characterization of liposomes was performed by transmission electron microscopy (TEM) (Fig. 4a) and NP stability was determined by measuring the ζ-potential with values >|30 mV|. Balbino et al. [153] evaluated the performance of two microchannels in the production of plasmid DNA/cationic liposome complexes. Two channels were compared, a MHF design and a serpentine microchannel with baffles (contraction–expansion regions) (Fig. 4b). The organic phase was composed of egg phosphatidylcholine (EPC), 1,2-dioleoyl-snglycero-3-phosphoethanolamine (DOPE), and 1,2-dioleoyl-3-trimethylammonium-propane (DOTAP) with (50/25/25% molar) in ethanol. At an FRR of 5, liposomes produced in the serpentine mixer had an average size of 118 nm and a PDI of 0.2, while the MHF design had an average size of 138 nm and a PDI of 0.35.

Maeki et al. [105] investigated the influence of flow parameters and herringbone structure height (31 μm and 11 μm) on liposome size. The microchannel with 31 μm structures was able to produce liposomes (1-palmitoyl-2-oleoyl-sn-glycero-3-phosphocholine (POPC) 5 mg/ml in ethanol) less than 100 nm (30–60 nm) across all FRR (3–9) and TFR (5–500 μL/min) outperforming the 11 μm design. Kimura et al. [154], demonstrated the precise control of LNP size in a 2D-baffle PDMS mixer device (invasive lipid NP production device (iLiNP)). POPC at a concentration of 10 mg/ml in ethanol and saline solution were injected into the device at flow rates ranging from 50 to 500 μL/min and a FRR of 3–9. Where LNP size was tuned at a 10 nm interval from 20 to 100 nm by changing the flow conditions and channel dimensions. In addition, they showed the scalability and high drug encapsulation ability (> 90%) of their design.

Aghaei et al. [141] synthesized methotrexate (MTX) loaded liposomes in a double flow focusing microfluidic device. MTX is a cytotoxic drug for the treatment of cancer and other diseases. Since MTX is hydrophilic, it was incorporated into the aqueous phase with deionized water, whereas the organic phase included EPC, cholesterol, and PEG-DSPE at 5 mM in ethanol. Non-pegylated MTX loaded liposomes had an average size ranging from 90 to 230 nm and a PDI < 0.32, while the pegylated loaded liposomes had sizes ranging from 118 to 250 nm and a PDI < 0.23 with an encapsulation efficiency > 60%. Characterization including nano-structural morphology and ζ-potential are presented in (Fig. 4c).

Erfle et al. [157] utilized an axis symmetric glass microchannel with five inlets for the production of LNPs. The mixing mechanism is based on the segmentation of the continuous flow with a gaseous phase, resulting in a periodic gas–liquid flow known as Taylor flow (Fig. 4d). The central inlet injects nitrogen gas while the remaining four inlets pump the organic phase (5 mg/ml castor oil and polysorbate 80 at 2.5 mg/ml) and deionized water. With 10% organic phase and a TFR from 31 to 130 μL/min, LNP size ranged from 70 nm (130 μL/min) to 90 nm (31 μL/min) and a PDI of 0.12–0.15, respectively. In another work by the same group [155], the segmented flow glass microchannel was compared to a high pressure micromixer and a staggered herringbone micromixer (NanoAssemblr™ platform) in the preparation of solid lipid NPs from castor oil and glycerol monooleate in ethanol. The herringbone and high-pressure mixers resulted in the smallest NP size at the highest flow rates (36 nm with castor oil at 10 mL/min, and 73 nm with glycerol monooleate at 101 mL/min).

A group led by Yvonne Perrie has published numerous articles on the synthesis of liposomes via the commercially available NanoAssemblr™ benchtop staggered herringbone microchannel [88, 158–163]. For example, in [88], they demonstrated for the first time the possibility of loading hydrophilic and lipophilic drugs into liposomes simultaneously. DSPC phospholipid was found to be the most sensitive in term of variation in the aqueous and alcohol solvents. For drug loading, the hydrophilic drug (metformin) was added to the PBS and the lipophilic drug (glipizide) was added to the lipid and alcohol solution. It was found that the encapsulation efficiency was 40% and 25% for the lipophilic and hydrophilic drugs, respectively. Moreover, in [163], they compared the utilization of methanol, ethanol, and isopropanol as the organic solvent in liposome synthesis via the staggered herringbone microchannel. Similarly, López et al. [164] compared these alcohols with Transcutol for the synthesis of liposomes in a PDMS microchannel with a CEA design. Transcutol is an organic solvent used in commercial skincare products and dietary supplements. In its pure form, it is safer to use than methanol and less polar than isopropanol. The use of Transcutol produced liposomes with the smallest size (90 nm) and the second lowest reported PDI 0.18, behind methanol. In addition, it showed greater liposome stability in synthesis temperatures ranging from 25 to 70 °C and after 50 days of storage.

Conversely to the previously reported works where the majority of microchannels were fabricated from glass or PDMS, other researchers explored polymer microfluidic fabrications via cheaper and simpler methods such as 3D printing and laser ablation [83].

Aranguren et al. [156] utilized laser cutting for the fabrication of poly(methyl methacrylate) (PMMA) serpentine microchannels (Fig. 4e). Two PMMA devices were considered: a two-layer design where the channel was engraved on one side and a three-layer design where the laser cuts through the 3 mm PMMA sheet. Similar results were obtained for both configurations, whereas the FRR increased from 2 to 5, at a constant TFR of 5 mL/min, size decreased from 250 to 188 nm (PDI 0.5–0.2). Similarly, Ballacchino et al. [165], investigated the applicability of 3D printing using fused deposition modeling (FDM) to fabricate micromixers for the synthesis of curcumin loaded liposomes. Liposomes were formulated from DMPS and cholesterol, 1 mg/ml in ethanol and PBS. Four designs were printed, including a zigzag design and a half-moon geometry. The half-moon design produced the smallest NPs ranging from 193 to 250 nm and 0.215–0.259 PDI (TFR 1–3 mL/min and 1:1 FRR).

##### Polymer NPs

**Fig. 5:**
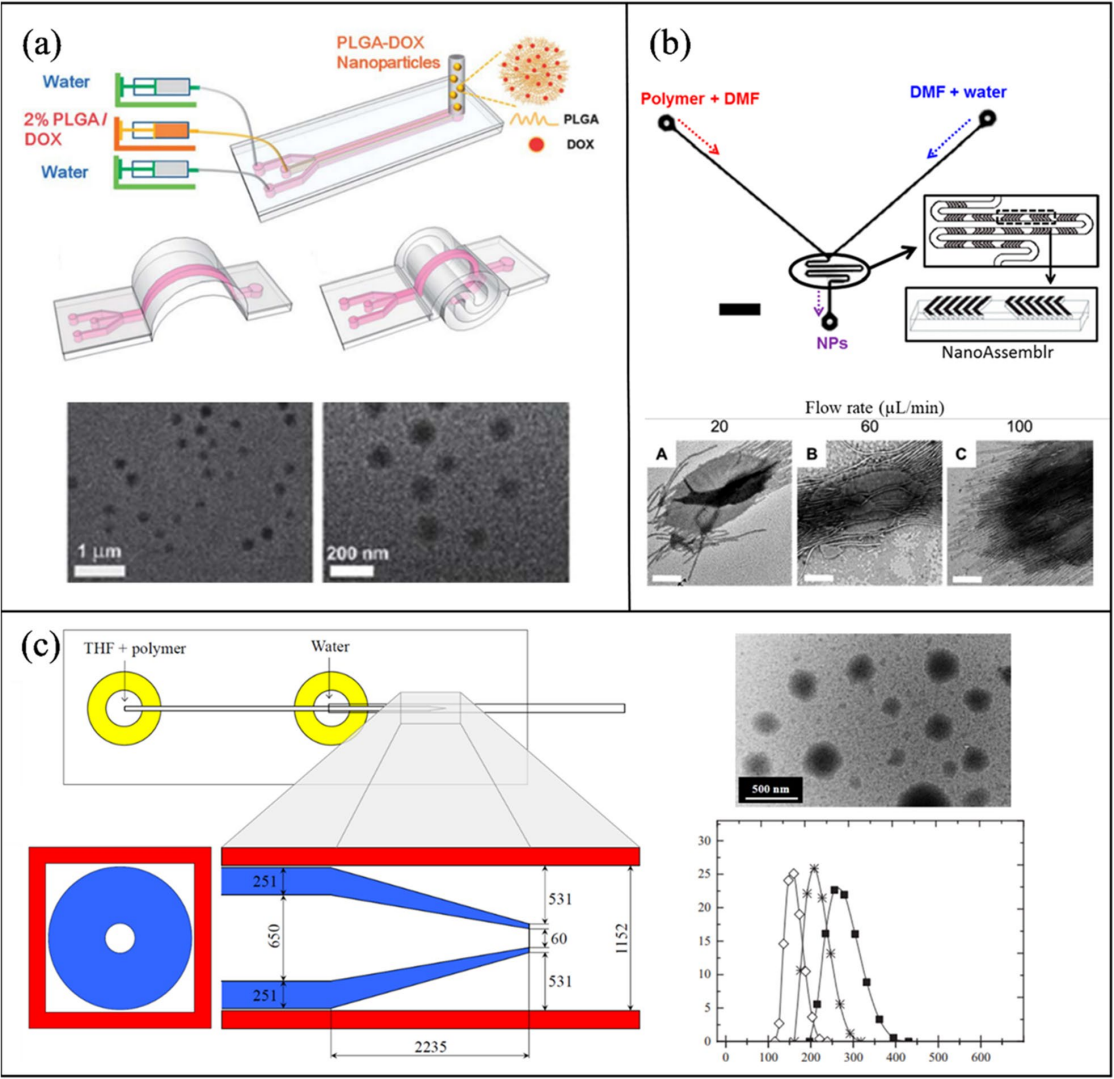
Passive microfluidic mixers for polymer-based NP synthesis. **a** A versatile “origami” microchannel for the fabrication of PLGA-DOX NPs [166]. **b** PCL-b-PEO synthesis in a staggered herringbone mixer (NanoAssemblr™) and a segmented flow-based mixer*.* Reprinted with permission from [168]. Copyright 2016 American Chemical Society (**c**) A concentric glass capillary within a square capillary microchannel for PCL and PLA production. Reproduced with permission from [140]. Copyrights © 2015 Elsevier

Sun et al. [166] developed a microfluidic chip with a PDMS microchannel and base for the synthesis of doxorubicin (DOX) loaded PLGA NPs. The flexible device was termed “origami” because of its ability to be folded manually into several configurations. The organic solution was prepared by dissolving 2% PLGA-DOX in dimethylformamide (DMF) and trifluoroethanol (TFE). Different origami configurations were compared, i.e., straight, arc, and spiral. The straight channel produced PLGA-DOX NPs in the size range of 100–230 nm, while the other designs resulted in smaller and more uniform NPs (75–100 nm, PDI < 0.13). TEM images of NPs produced in a 3D spiral channel are shown in Fig. 5a. Solorzano et al. [167], were able to continuously synthesize cyclosporine (drug used after organ transplants) encapsulated PLGA NPs in an interdigital micromixer with a mean particle size of 211 ± 62 nm and an encapsulation efficiency of 91%.

Abdelkarim et al. [169] tested ten different microfluidic designs and evaluated their performance on the physicochemical properties of PLGA NPs, where 5 mg/ml of PLGA was dissolved in DMF. The parameters under investigation were channel length, FRR, aspect ratio, number of interfaces, and curvature. Channel length did not have a significant effect on size or PDI. When the FRR was increased from 1:1 to 10:1, particle size decreased from 265 to 93 nm. This occurs because of the decrease in the organic stream size, which shortens the diffusion distance. Increasing the aspect ratio (height: width) at the same cross-sectional area, from 1:10 to 4:1 reduced the NP size from 137 to 71 nm at a TFR of 132 μL/min and 5 FRR. Increasing the number of interfaces between the streams (more inlets) increases the diffusion area between the phases and results in a reduced size (130–92 nm). Finally, two spiral designs with different curvatures were compared: 2.23 mm^−1^ and 0.45 mm^−1^. Reducing the curvature from 2.27 to 0.45 mm^−1^, increased the Dean Flow vortex formation and reduced the PLGA NP size from 84.86 to 69.3 nm. Thus, it was shown that the aspect ratio, number of inlets, and curvature are important in tuning NP size without the extra dilution as in the FRR case.

Morikawa et al. [170] utilized the NanoAssemblr™ system to synthesize curcumin-loaded PLGA NPs and study the effects of different stabilizers. The organic phase contained 10 mg/mL of PLGA and 1 mg/mL of curcumin in acetone, while the aqueous phase had either sodium cholate, Tween 80, or Polyvinyl alcohol (PVA) in water. The 1% PVA showed the best NP synthesis at 200 nm, 0.13 PDI, and an encapsulation efficiency of 18%. Moreover, the addition of 5% PEG reduced the particle size to 120 nm and increased the encapsulation efficiency to 50%.

Xu et al. [168], compared the formation, morphology, and crystallinity of PCL-block-poly(ethylene oxide) (PCL-*b-*PEO) NPs in a staggered herringbone mixer (NanoAssemblr™) (Fig. 5b (i)) and a PDMS/glass segmented flow-based mixer (Taylor flow) (Fig. 5b (ii)). The segmented flow mixer had four inlets, (1) DMF, (2) DMF and polymer, (3) DMF and water, and (4) argon gas. Both experiments were carried out at 20–100 μL/min and 1:1 FRR. Where sizes in the range of 15–21 nm were achieved in both mixers. Othman et al. [140] utilized an inner tapered round glass capillary within an outer square capillary assembled to form a microfluidic mixer. Rapid diffusion occurs due to the 3D exposure of organic solvent to water. A solution of PCL or PLA in THF and the aqueous phase of Milli-Q water were injected into the coaxial and square glass capillaries respectively to obtain polymeric nanoparticles. TEM and DLS were used to characterize the morphology, and size and PDI, respectively (Fig. 5c).

#### Inorganic NP synthesis

**Fig. 6:**
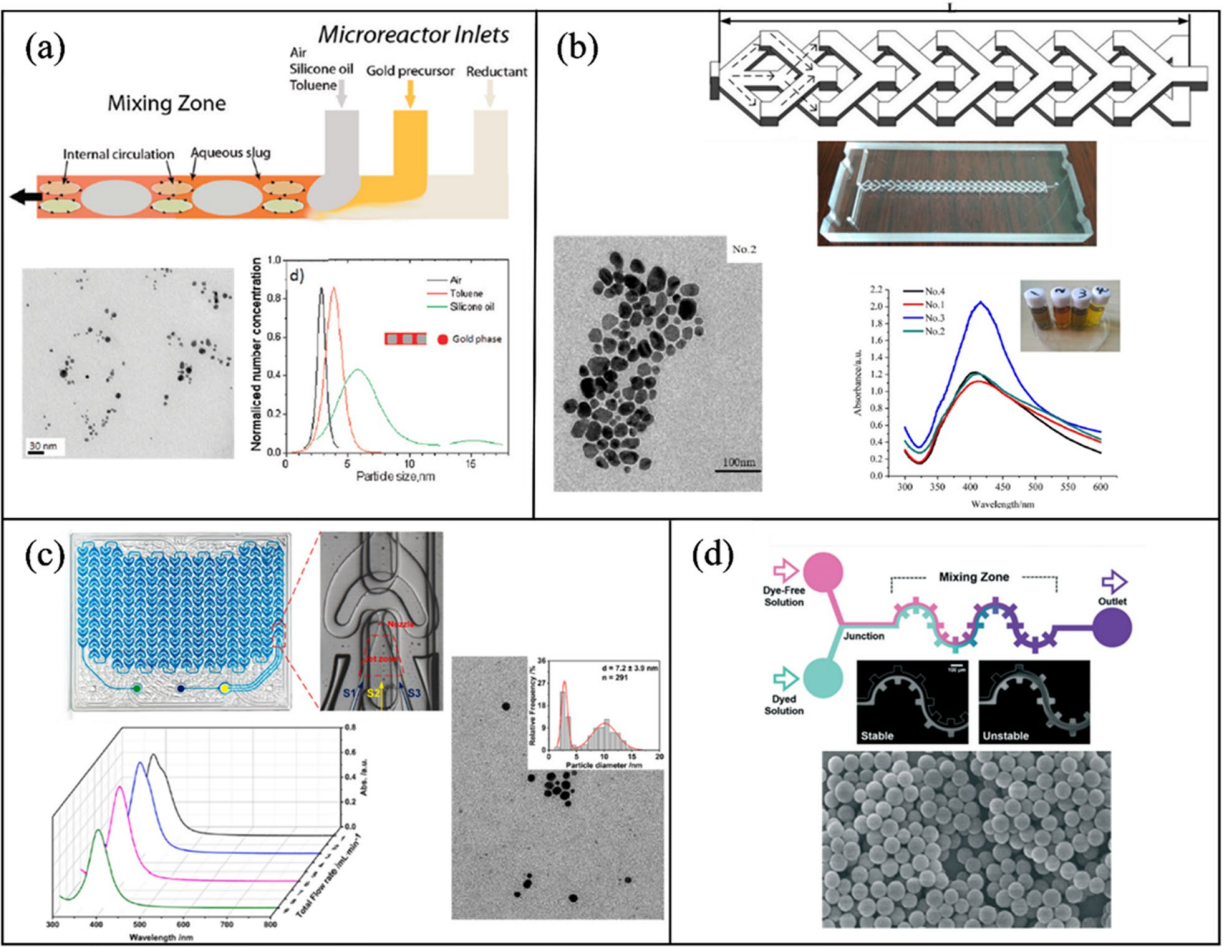
Passive microfluidic mixers for inorganic NP synthesis (**a**) AuNPs synthesis in a silicon segmented flow microchannel. Reprinted with permission from [171]. Copyright 2012 American Chemical Society. **b** A double layer Y-shaped split and recombination micromixer for AgNPs fabrication [174]. **c** A curved split and recombination (Corning AFR) for the synthesis of AgNPs. Reproduced with permission from [175]. Copyrights © 2021 Elsevier. **d** Silica NPs production in a gear-serpentine microchannel [176]

Cabeza et al. [171] designed a three-inlet silicon microchip for the synthesis of AuNPs employing the segmented flow mixing mechanism. A silicon wafer was etched with a spiral design and anodically bonded to a glass slide. The platform was heated to 100 °C where streams of sodium borohydride (NaBH_4_) (reducing agent), gold precursor chloroauric acid (HAuCl_4_), and toluene (separating fluid) where injected in to the microchannel to form the segmented flow (1:10:1). An increase in microchannel residence time resulted in the broadening of the size distribution from 3.8 ± 0.3 nm (10 s) to 4.9 ± 3 nm (40 s). AuNPs characterization was determined by TEM imaging and UV–Vis spectroscopy (Fig. 6a). Utilizing the same gold precursor and reducing agent, Lazarus et al. [172] prepared AuNPs in a PDMS serpentine microchannel with good control over size and morphology (4.38 ± 0.53 nm). Sarsfield et al. [173], synthesized AuNPs in a reverse-staggered herringbone PDMS micromixer. Several parameters, such as the ratio of the sodium citrate to HAuCl_4_, and the pH of the HAuCl_4_ solution, were considered to study the effect on AuNP size and size distribution.

Zhang et al. [177] utilized a PMMA serpentine microchannel for AuNP preparation. 4.5–7 nm NPs were produced with optimum conditions at 5:3 FRR, 0.2 mL/min, and 100 °C.

Other researchers utilized passive micromixers to produce different types of inorganic NPs. Liu et al. [174] studied the effects of TFR, reducing agent concentration, and PVP on the synthesis of silver NPs (AgNPs) in a double-layer Y-shaped split and recombination micromixer (Fig. 6b). Increasing the reductant concentration increased the average size of AgNPs from 20 ± 6.7 to 31.43 ± 4.47 nm, as more silver atoms accumulate in the crystal growth stage. In addition, increasing the TFR from 6.7 to 810 μL/min varied the size slightly from 29.9 ± 5.3 to 30.5 ± 4.82 nm because of the good mixing performance of the double layer micromixer. Thiele et al. [178] synthesized 4.7 ± 0.6 nm AgNP seeds in a T-mixer for the later production of triangular NPs which was confirmed by AFM and SEM imaging. Yang et al. [175] evaluated the performance of the commercially available Corning AFR (Lab reactor module) on the synthesis of AgNPs. The Corning AFR is glass based with two inlets and relies on the split and recombination mixing mechanism with a curved design. NaBH_4_ and Ag precursor were used at a TFR of 9 mL/min and 2 FRR, resulting in a NP size of 4.6 ± 1.8 nm. Figure 6c shows UV–Vis spectroscopy data for AgNPs at different flow rates and a TEM image at 1 mL/min. Baki et al. [179] studied the effect of reaction temperature and residence time on the physicochemical properties of magnetic single-core iron oxide NPs. Where tunable NP sizes (20–40 nm) was achieved with high quality magnetic properties by varying these two parameters. Thu et al. [180] synthesized 10 nm magnetite NPs in serpentine PDMS microchannel, where a mixture of iron (II) and iron (III) acidic, and sodium hydroxide were used as precursors for the production.

Hong et al. [176] presented a novel design which combines a serpentine geometry with rectangular sections perpendicular to the channel, forming a gear shaped design (Fig. 6d). Silica NPs were synthesized at 370.3 nm at 1 mL/h with over 90% mixing efficiency.

#### Hybrid NP synthesis

**Fig. 7:**
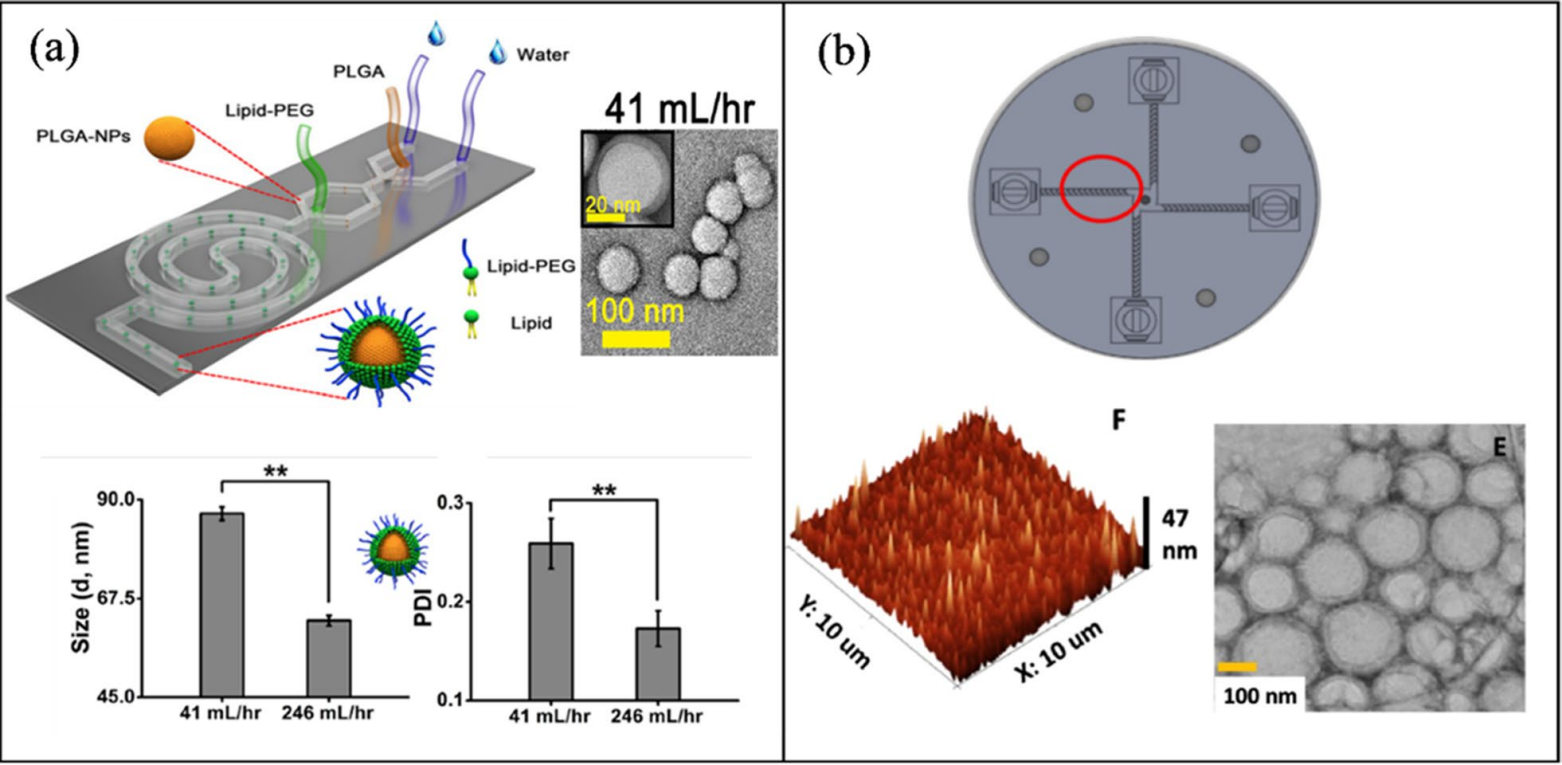
Passive microfluidic mixers for hybrid NP synthesis (**a**) PLGA-lipid NPs in a spiral microchannel (open access) [182]. **b** A staggered herringbone patterned multi-inlet vortex mixer for the synthesis of PLGA-lipid NPs (open access) [183]

Valencia et al. [181] demonstrated the synthesis of PLGA core lecithin shell NPs, and quantum dots (QDs) coated lecithin in a single step via a PDMS Tesla structure micromixer. Where the lecithin and DSPE-PEG (8.4:1.6 by mol) were incorporated into the aqueous solution and the PLGA (1 mg/mL) dissolved in acetonitrile. At a FRR of 10:1 and a TFR of 50 μL/min, hybrid organic NPs were produced with an average size of 40 nm and a mixing time of 10 ms. Similarly, QD dissolved in THF at a concentration of 0.5 mg/mL produced organic–inorganic hybrid NPs with an average size of 60 nm. Feng et al. [182], employed a two stage microfluidic device for the synthesis of a PLGA (core)/lipid (shell) NP. Initially, PLGA (1% in TFE and DMF) organic solution was injected with an aqueous phase to form the polymer core, followed by a second inlet stage delivering the lipid phase, DPPC, DSPE-PEG and cholesterol (total 2.94 mg/mL) in ethanol. Followed by a spiral channel to enhance mixing and assemble the lipid shell over the polymeric core. At a TFR of 41 mL/h (246 mL/h) and a FRR of 80:1, the hybrid NP had an average size of 86.81 ± 1.5 nm (62.5 ± 1.18 nm) and a PDI of 0.259 (0.173). Figure 7a shows size and PDI measured by DLS along with NP morphology characterization by TEM. Bokare et al. [183] utilized a 3D printed multi-inlet vortex mixer (MIVMS) for the production of lipid-polymer NPs (LPHNPs) (lecithin-PLGA) (Fig. 7b). A comparison was made between a staggered herringbone patterned MIVMS and a regular MIVMS, and a 3-inlet MHF PDMS microchannel. The herringbone patterned MIVMS produced the smallest hybrid NPs with an average size of 60–70 nm and PDI < 0.12 at 12 ml/min and showed better reproducibility in comparison to the other microchannels. The lipid-polymer NPs was characterized by DLS, TEM, and atomic force microscopy (AFM).

Larrea et al. [184] designed a microfluidic system comprised of two consecutive slit-interdigital micromixers to synthesize gold loaded PLGA NPs. Initially, the organic phase (13.5 mL/h) is mixed with an aqueous phase containing HAuCl_4_, sodium citrate, and water (4.5 mL/h). The effluent is then injected into the second interdigital mixer to mix with sodium cholate and Milli-Q water at 36 mL/h. After encapsulation, the loaded gold is heated to 45 °C and reduced. Thus, they were able to undergo the reduction of AuCl_4_^−^ ions while encapsulated, which resulted in a 100% encapsulation efficiency. In addition, increasing the TFR from 36 to 54 mL/h decreased the size from 568 to 192 ± 58 nm. Ohannesian et al. [185] presented the synthesis of polymer-10 nm magnet metal oxide NP hybrid in a microfluidic reactor. Where iron sulfate/iron nitrate precursors where mixed with sodium hydroxide/dextran to produce superparamagnetic iron oxide NPs coated with dextran (long chain polymer). Similarly, Ding et al. [186] produced superparamagnetic iron oxide core encapsulated in PMMA NPs with sizes ranging from 100 to 200 nm.

Al-Ahmady et al. [187] synthesized a metal–organic (AuNPs loaded liposomes) NP hybrid in the commercially available Asia MF 320 system (Syrris, Royston, UK). Due to the hydrophobic nature of AuNPs, they were added to the organic phase in methanol. Empty liposomes were produced with an average size of 100 nm, while the AuNPs hybrid had a size range of 130–260 nm which were analyzed and confirmed by AFM. Di Santo et al. [188] utilized the NanoAssemblr™ (staggered herringbone) benchtop to fabricate graphene oxide-cationic lipid NPs (modification confirmed by AFM), and Rohra et al. [189] reported on the synthesis of AuNPs-metal–organic framework (MOF) in a split and recombination channel. The micromixer was made of acrylic sheets and fabricated by computer numerical control (CNC). The MOF was made of zeolitic imidazolate framework-8, a class of MOF that is formed by the self-assembly between imidazolate and Zn^2+^. Wang et al. [190] synthesized hybrid NPs composed of metal alloy cores and metal oxide shells in a multistep procedure involving programmed microfluidics and batch cooling processes. Such hybrid NPs include Fe_(1-x)_Zn_x_ (core) Zn_(1-y)_Fe_y_O-(OH)_z_ (shell), an iron-zinc-based NP.

### Active micromixing

Active mixing methods rely on external forces to disturb the flow and induce chaotic advection to increase the contact area between the different fluids, thus enhancing the mixing quality and time. Depending on the applied force, active micromixing can be further classified as acoustic [193–199], electrical [200, 201], thermal [202, 203], and pressure [124, 204, 205] field driven methods.

#### Background on active mixing methods

This section presents a brief background on the various active mixing methods utilized for NP synthesis.

##### Acoustic mixing

**Fig. 8:**
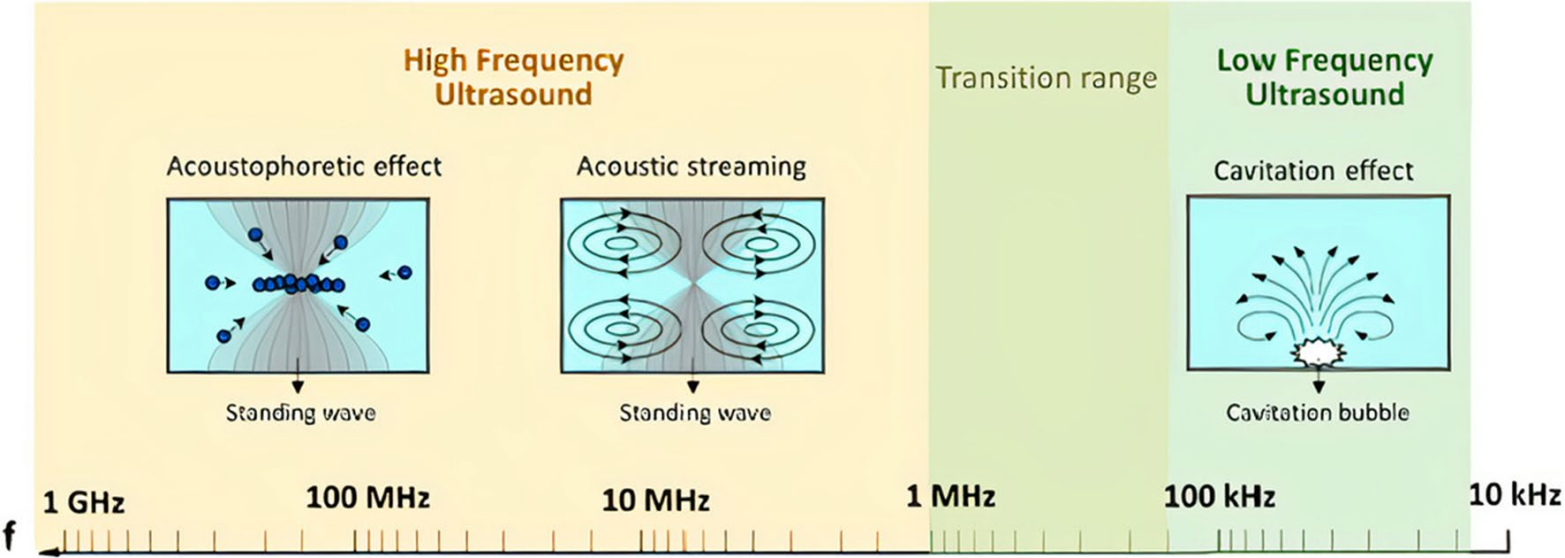
Acoustic phenomena associated with high and low frequency ultrasound (open access) [206]

**Fig. 9:**
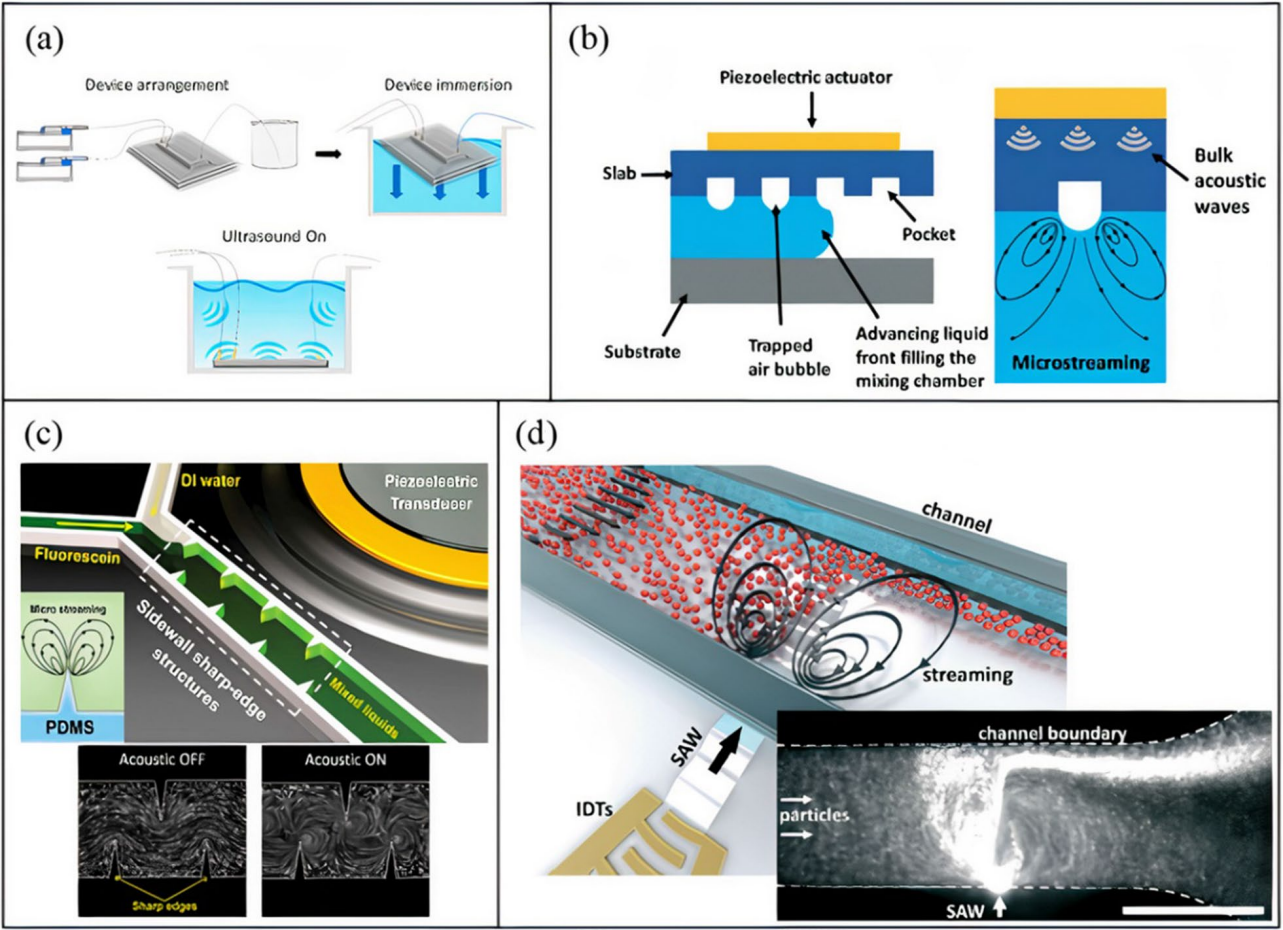
Acoustic mixing methods. **a** Ultrasonic bath mixing (open access) [128], **b** trapped bubble mixing (open access) [219]), **c** sharp edge mixing (open access) [220], and **d** SAW acoustic streaming (open access) [221]

Acoustic-based micromixing is a versatile method of active mixing where it encompasses the application of frequencies ranging from 1 kHz to 1 GHz [206, 207]. One of the most common methods of generating acoustic waves relies on the inverse piezoelectric effect, where electrical signals are transformed into mechanical disturbances. Depending on the applied frequency, the propagating acoustic wave induces different physical mechanisms in the fluid (Fig. 8). At frequencies below 200 kHz, microbubble cavitation is the prominent physical phenomenon that occurs [208], which can enhance mixing [199, 209, 210], and prevent clogging and NP aggregation [211]. During acoustic cavitation, pre-existing and newly formed microbubbles in the liquid medium oscillate vigorously with the applied acoustic pressure. The microbubbles coalesce, grow, and undergo shape and volume oscillations. As the bubble size increases and reaches it resonance size, transient cavitation occurs, resulting in the collapse of the bubble and the generation of strong turbulence (cavitation microstreaming), liquid jets, and shockwaves [212]. These effects enhance mixing by disturbing the laminar flow in microchannels, inducing vortices and chaotic advection. Acoustic cavitation is dominant in low ultrasonic frequencies because of the low acoustic power threshold at the applied frequency. The power threshold increases for higher frequencies, which is why cavitation is not observed at megahertz scales [206]. Acoustic cavitation in microchannels can be realized by several methods including immersing microchannels in ultrasonic baths [152, 162, 166] (Fig. 9a), and actuation by piezoelectric transducers bonded to glass or silicon substrates [213, 214].

Another form of low frequency acoustic mixing is the interaction of the applied acoustic waves with embedded structures in microchannels. There are two main methods of achieving such mixing: trapped bubble and sharp edge oscillations. Both methods rely on the acoustic actuation of a piezoelectric transducer bonded to a silicon or glass substrate via a PDMS microchannel [94, 215–218].

In trapped bubble oscillation, an aqueous solution is initially injected into a PDMS microchannel with microcavities. As the fluid flows, gases are trapped in the cavities due to surface tensions, forming bubbles [222]. Bubble size can be tuned by changing the size of microcavities. As the piezoelectric transducer is driven, bubbles start to oscillate (volume and shape oscillations) and, due to the frictional forces and viscous attenuation of acoustic waves in the fluid, bulk fluid motion arise around the bubbles (Fig. 9b) [223]. When the applied frequency matches the natural frequency of the oscillating bubble, maximum oscillation amplitude occurs [198]. The induced circulatory motion is effective in disrupting the laminar flow and enhancing mixing. Conversely to the acoustic cavitation discussed earlier, in trapped bubble oscillation, bubble size is relatively larger and do not collapse as the required power threshold is not reached, which is also not the purpose of such devices. However, the bubbles can destabilize due to long term actuation of the acoustic transducer and heating of the device, which results in expansion of bubble volume. Therefore, it is vital to keep the channel temperature in check to avoid undesired change in bubble size that can also affect the resonant actuation frequency of the device.

Alternatively, oscillating sharp edges, which are structures protruding inside the microchannels, and generally composed of either PDMS [216] or silicon [218] (same as the base material), are relatively immune to such limitations. Upon actuation of the piezoelectric transducer, the sharp edges will oscillate with the applied frequency and generate a pair of counter-rotating vortices at the tip (Fig. 9c) [216]. Similar to the oscillating bubbles, the generated acoustic streaming can break the interface of the laminar flows and enhance mass transfer and mixing. However, there are two main operational differences between the two methods: (i) with trapped bubble oscillation, the applied frequency must coincide with the natural frequency of the bubble, (ii) whereas with the sharp edge design, the piezoelectric transducer is operated at its own resonance frequency for optimum mixing conditions. Moreover, optimization of sharp edge mixing includes many parameters, including tip angle, sharp edge size and length, density, pattern, in addition to the applied frequency, number of objects, voltage, and flow rate (which are common with bubble oscillation). For example, smaller tip angles result in stronger acoustic streaming [224], larger sharp edges (to a certain size) are better for mixing, and higher voltages result in larger vibrational amplitudes and stronger acoustic streaming [216].

At higher frequencies (> 1 MHz), cavitation is not observed, but acoustic radiation forces and acoustic streaming flows are induced in microchannels, which can be used for microparticle manipulation and mixing [221]. The acoustic streaming flow phenomena occurs because of gradients in the acoustic field brought about by the scattering, absorption, and dampening of the acoustic waves when they interact with the fluid and channel structure [225]. To add to the versatility of acoustic-based mixing, at megahertz scales, different mechanisms and materials are used to produce acoustic waves. One of the most common methods of acoustic actuation at these scales is the utilization of Surface Acoustic Waves (SAW) also known as Rayleigh waves. SAWs are acoustic waves (10–100 μm in wavelength) propagating along the surface of an elastic medium with a penetration depth into the material about five times the wavelength [226]. SAW mixing platforms consist of one or multiple interdigitated transducers (IDTs), which are comb-like metallic electrodes patterned on a piezoelectric substrate, and a PDMS channel (Fig. 9d). IDTs are fabricated using standard photolithography and wet etching, and have a resonance frequency dependent on electrode width, interelectrode gap, and the speed of sound in the piezoelectric substrate. One of the most commonly used piezoelectric materials is lithium niobate (LiNbO_3_), which can be further classified depending on the cutting angle during the fabrication process. With, 127.68° Y-X-axis-rotated cut, X-propagating LiNbO_3_ is the most widely used substrate [80, 137, 193, 221, 227–229] because of its high electromechanical coupling coefficient [230]. When an alternating current is applied to an IDT at the resonance frequency, the substrate undergoes mechanical displacement due to the presence of an electric field and the piezoelectric effect [231]. A SAW is generated at the IDT and travels along the surface of the piezoelectric material until it encounters the PDMS channel, where it leaks into the microchannel, generating pressure fluctuations within the fluid [225]. Due to the speed of sound difference between the fluid medium *v*_*f*_ and the piezoelectric substrate *v*_*s*_, the leaked waves enter the fluid at an angle known as Rayleigh angle (*θ*_*R*_). This angle is determined by Snell’s law: sin (*θ*_*R*_) = *v*_*f*_/*v*_*s*._ As the leaky SAW propagates across the microchannel, it is attenuated by viscous dissipation, creating a steady momentum flux in the direction of wave propagation, which results in steady fluid motion in the form of acoustic streaming flow. The induced steady motion is known as Eckart streaming which occurs when the channel width is greater than the acoustic wavelength [229]. The generated acoustic streaming is used to disturb the laminar flow and enhance mixing.

##### Electrical/thermal mixing

**Fig. 10:**
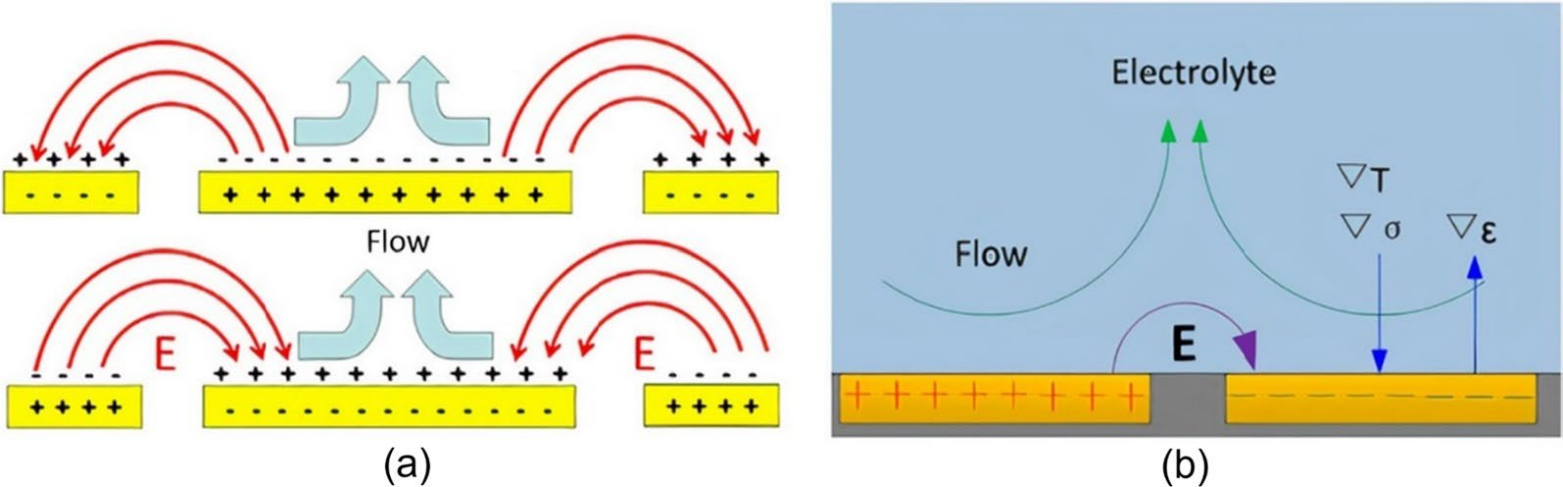
**a** Electrohydrodynamic mixing (open access) [236], and **b** alternating current electrothermal mixing. Reproduced with permission from [237]. Copyrights © 2017 John Wiley & Sons Ltd

Electrical micromixers are typically embedded with electrodes within the microchannel and, upon DC or AC voltage excitation, fluid motion is induced. Electrical mixing in microchannels can be achieved in various ways, such as electrohydrodynamics (EHD), which relies on the fluids’ distinct electrical properties, and alternating current electrothermal (ACET) and direct current induced thermal buoyancy convection (DCIBC) arising from joule heating [232]. EHD mixing develops from flow instabilities at a fluid–fluid interface when an electrical stress is applied (Fig. 10a). Electrical stresses are generated at the interface due to the sharp discontinuity in electrical properties (conductivity or permittivity) of the fluids in the presence of an electric field [233]. Deionized water and ethanol are examples of such fluids, where they have similar conductivities but distinct permittivities (*ε*_water_ = 80, *ε*_ethanol_ = 24.5) [95]. EHD fluid actuation is strongly affected by the AC frequency, amplitude, and the electrical properties of the fluids [234]. On the other hand, ACET involves inducing micro-vortices in the channel due to the interaction of a temperature gradient in the fluid and a non-uniform AC electric field (Fig. 10b) [235]. A temperature gradient in the fluid arises because of Joule heating, which causes a gradient in the electrical properties (conductivity and permittivity) of the fluid. This variation in electrical parameters generates an electrical body force on each fluid medium, resulting in fluid motion and vortices [202]. The ACET is a function of temperature gradients, higher fluid conductivities, and electrode geometry, where asymmetric electrodes generate non-uniform joule heating and efficient mixing [203].

#### Acoustic NP synthesis

Table 3 outlines the active mixing microchannels and mechanisms for NP synthesis.

**Table 3:** Microfluidic synthesis of nanoparticles by active mixing methods

Nanoparticle type	Mixing mechanism	Microchannel	Voltage (Vpp)	Size (nm)	PDI		Organic/precursor Concentration	FRR	TFR	ME	MT	Year	Ref
*Acoustic*													
PLGA	Piezoelectric transducer (205 kHz)-acoustic streaming	Glass capillary	20	65	0.08		30 mg/mL	0.3	8 μL/min	90%	103 ms	2022	[117]
PLGA	Lotus shaped cantilever (8) mixer (680 kHz)-acoustic streaming	Silicon/PDMS	–	52.2	0.44		1 mg/mL	1:1	1400 μL/min	80%	2 ms	2022	[218]
Budesonide	Square cross section membrane vibration (177.6 kHz)-acoustic streaming	Silicon/PDMS	200	135.7	0.044		0.2 mg/mL	1:4	10 μL/min	–	3 ms	2018	[250]
PLGA	PDMS sharp edges, 13 pairs (4 kHz)-acoustic streaming	Glass/PDMS	30	64.7 ± 0.7	0.13		10 mg/mL	–	10 μL/min	100%	54 ms	2019	[220]
PLGA	Tesla structures/PDMS sharp edges (4.9 kHz)-acoustic streaming	Glass/PDMS	56	64.5	0.062		10 mg/mL	3:7	2000 μL/min	100%	–	2020	[241]
PLGA	Bubbles/sharp edges (74.2 kHz)-acoustic streaming	Glass/PDMS	40	40	0.072		1.25 mg/mL	1:4	20 μL/min	88%	~ 1 ms	2019	[94]
Liposomes	Bubbles/sharp edges (74.2 kHz)-acoustic streaming	Glass/PDMS	40	80	0.125		1 mg/mL	1:6	60 μL/min	88%	~ 1 ms	2019	[94]
SLN	Piezoelectric transducer (500 kHz), acoustic cavitation	Silicon/Glass/PDMS	100	80 ± 2	0.34		0.4 mg/mL	3:25	56 μL/min	–	–	2021	[213]
PFC loaded PLGA	Sonication flow cell	–	–	160	0.2		–	–	37.5 mL/min	–	–	2020	[242]
bPEI polyplexes	SAW (81.2 MHz)-acoustic streaming	LiNbO3/PDMS	5.6	110.8 ± 0.65	0.283		0.12 mg/ml (bPEI), 0.156 mg/ml (pDNA)	–	2.5 μL/min	100%	189 ms	2016	[239]
AuNP	Rotary SAW mixing, 4 IDT (50 MHz)	LiNbO3/PDMS	35	21.32 ± 4.92	-		1 mM (HAucl4)	–	–	–	–	2018	[249]
PLGA	PDMS sharp edges, 12 pairs (4 kHz)-acoustic streaming	Glass/PDMS	38	101	0.17		30 mg/mL	1:10	55 μL/min	100%	4 ms	2021	[245]
Protein NP	Star shaped cavity (960 kHz)-acoustic streaming	Silicon/PDMS	1	149.6	0.38		0.3 mg/mL	–	500 μL/min	85%	6 ms	2020	[240]
Exosome membrane/PLGA	Spiral channel/sonication (80 kHz)	Glass/PDMS	–	177.4	0.193		5 mg/mL	7:80	2900 μL/min	–	–	2019	[209]
Cancer membrane/PLGA	Spiral channel/sonication (80 kHz)	Glass/PDMS	–	172.3	0.222		5 mg/mL	7:80	2900 μL/min	–	–	2019	[209]
Lipid/PLGA	Spiral channel/sonication (80 kHz)	Glass/PDMS	–	157.2	0.195		10 mg/mL	7:80	2900 μL/min	–	–	2019	[209]
AgNP	SAW (9.2 MHz)-acoustic streaming	LiNbO3/PDMS	21	45 ± 11.5	–		0.1 M (AgNO3)	–	100 μL/min	100%	20 ms	2017	[248]
Budesonide	Star shaped cavity (1060 kHz)-acoustic streaming	Silicon/PDMS	1	80.53 ± 21.95	–		4 mg/mL	1:4	5000 μL/min	91%	4.1 ms	2020	[252]
DNA NP	Star shaped cavity (1060 kHz)-acoustic streaming	Silicon/PDMS	1	90	–		–	–	4000 μL/min	91%	~ 4.1 ms	2020	[252]
Liposomes	Ultrasonic bath (50–60 kHz)	Glass	–	66.27	–		3 mg/mL	1:12	3740 μL/min	–	–	2010	[199]
*Electric/Thermal*													
BaSO_4_	Alternating current electrothermal (1 MHz)	Glass/PDMS	12—20	75.3 ± 17.5	–		5 mM	–	0.9 μL/min	90%	–	2019	[202]
Cu_2_O	Direct current-induced thermal buoyancy convection	Glass/PDMS	8	680	–		0.2 M	–	18 μL/min	97.3%	–	2019	[253]
Liposomes	Electrohydrodynamic micromixing (1 MHz)	Glass/PDMS	10	126.7 ± 0.7	–		1 mg/mL	1:10	50 μL/min	60%	–	2020	[234]
Liposomes	Electrohydrodynamic micromixing (1 MHz)	Glass/PDMS	10	97	–		0.25 mg/mL	1:10	200 μL/min	–	–	2020	[95]
Co-Fe PBA	Alternating current electrothermal (1 MHz)	Glass/PDMS	22	231.1 ± 35.4	–		5 mM	–	–	90%	–	2020	[203]
*Other*													
SLN	Oscillator mixer (138.9 Hz)	–	–	93.4	0.184	100 mg/mL		1:9	60 mL/min	–	–	2015	[132]
AuNP	PZT micropump pulsed Mixing (300 Hz)	Glass/PDMS	–	21.6 ± 4.83	–	1 mM (HAucl4)		–	4 mL/min	97%	–	2015	[205]
AgNP	PZT micropump self-circulating mixing	PMMA/PET	80	10.31 ± 2.38	–	1 mM (AgNO3)		–	–	–	–	2019	[258]
AuNP	Soft wall vibration	Glass/PDMS	–	5	–	–		–	–	–	–	2015	[257]
AuNP	PZT micropump pulsed Mixing (200 Hz)	Silicon/Glass	100	42.7	–	0.48 mM (HAucl4)			2.4 μL/min	–	100 ms	2010	[204]
AuNP	Pneumatic micromixing (6 Hz)	Glass/PDMS	–	19	–	–		–	170 μL/min	95%	1 s	2010	[256]

*PDI* polydispersity index, *FRR* flow rate ratio, *TFR* total flow rate, *ME* mixing efficiency, *MT* mixing time

##### Organic NPs

Sonication in an ultrasonic bath is one of the oldest methods to prepare lipid nanoparticles [238]. Huang et al. [199] submerged a glass microchannel in a sonicator (50–60 kHz) to evaluate its effect on the preparation of liposomes. Overall, sonication reduced particle size from 150 to 50 nm, while 120 nm was the minimum diameter reached without sonication. Giraldo et al. [128] synthesized magnetoliposomes (MLP), which combine liposomes and nanostructured magnetic materials (magnetite) for drug delivery applications. A laser engraved PMMA microchannel was fabricated with a serpentine design. NPs were produced passively and actively by immersing the device in an ultrasonic bath (45 kHz). Passive mixing resulted in MLPs with a diameter of 344 ± 46 nm and a PDI of 0.33 ± 0.07, while with the addition of acoustic actuation, the diameter was reduced to 219 ± 1.8 nm with a PDI of 0.31 ± 0.03.

**Fig. 11:**
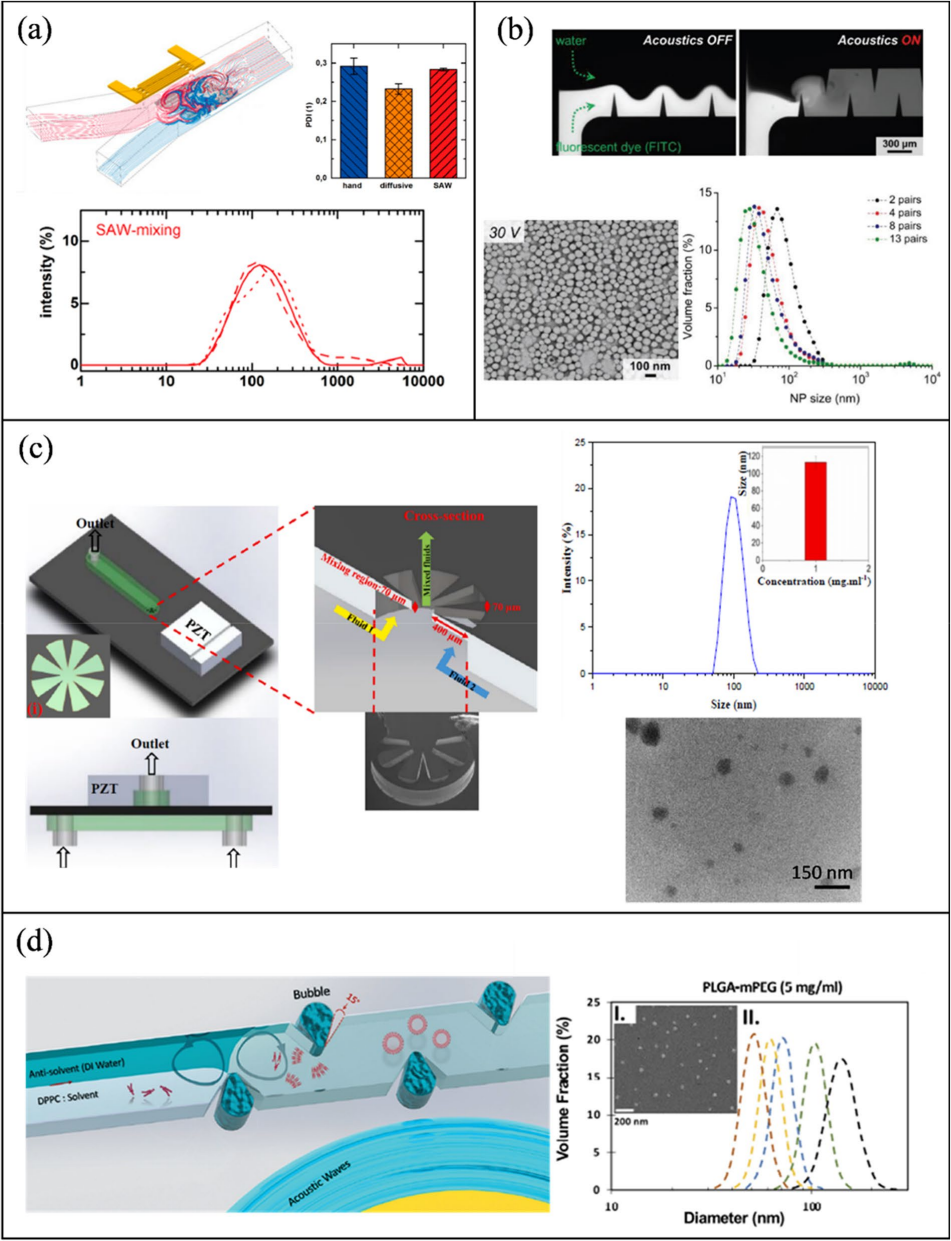
Acoustic micromixing for the fabrication of organic NPs (**a**) SAW-acoustic streaming for the synthesis of bPEI polyplexes and MNALP (open access) [239]. **b** PDMS sharp edges microstreaming for the production of PLGA NPs (open access) [220]. **c** PLGA NPs synthesis by silicon sharp edges (open access) [218]. **d** Combined oscillatory bubbles and sharp edges to fabricate PLGA NPs [94]

Westerhausen et al. [239] demonstrated another approach of acoustic mixing by utilizing surface acoustic waves (SAW) (Fig. 11a). The microchannel was made of PDMS on a piezoelectric substrate (lithium niobate) with a tapered interdigitated transducer (IDT) at a resonance frequency of 81.2 MHz. Two types of NPs were produced, bPEI polyplexes and mono-nucleic acid/lipid particles (MNALP). A comparison between their SAW platform and a MHF channel was conducted. The SAW microchannel produced bPEI polyplexes with a 55 nm diameter and 0.281 PDI and was shown to exhibit higher reproducibility, and smaller particles.

Bolze et al. [213] investigated the use ultrasound to prevent clogging due to the precipitation of lipids during solid lipid nanoparticle (SLN) production. The mixing chamber consisted of a piezoelectric disk and a silicon microchannel anodically bonded to a glass substrate. Each inlet diverges into ten channels to increase the surface area of mixing between the solvent (trimyristin, polysorbate80, and acetone) and anti-solvent (water). It was demonstrated that without acoustic actuation, lipid particles precipitated on the channel walls and the device started to leak after five minutes due to clogging. However, when the piezoelectric disk was operated (500 kHz and 100 *V*_pp_), NPs were synthesized for 4–7 h continuously with decrease in NP size to 80 nm ± 2 nm and a PDI of 0.34. Reduction in NP size was attributed to the matching of frequency with the device’s resonant frequency which leads to cavitation intensification and improved mixing.

Huang et al. [220] presented a PDMS acoustofluidic platform which combines acoustic actuation and sharp edges (Fig. 11b). To test their device’s effectiveness, multiple NP were synthesized, including PLGA and chitosan. They optimized the mixing performance by investigating different parameters, including frequency (4 kHz) and length of the sharp edge base (300 µm). By operating at the optimum parameters and increasing the number of sharp edge pairs from 2 to 13, the PLGA NP size decreased from 102.8 to 88.6 nm and the PDI improved from 0.21 to 0.13. By increasing the applied voltage to 30 V_pp_, a high mixing performance was achieved after the first pair of sharp edges in comparison to four pairs at 10 *V*_pp_. NP particle characterization was determined TEM, DLS, and ζ-potential measurement.

Pourabed et al. [218] developed a microfluidic system where a silicon wafer is sandwiched between two PDMS layers and glued to a piezoelectric disk (Fig. 11c). Fluids flow from the bottom of the channel through the silicon to the top PDMS layer, where the silicon substrate is patterned and etched to form sharp edges in a circular arrangement (lotus design). Conversely to the PDMS sharp edges in ref. [220], silicon sharp edges generate stronger body forces and acoustic streaming due to the higher stiffness and lower damping coefficient. At a frequency of 680 kHz, 52 nm PLGA NP were produced with a PDI of 0.44. Morphological characterization was done by TEM, and size and PDI were determined by DLS. In another effort by the same group [240], a similar system was presented but with a “star” design etched through the silicon wafer for the synthesis of protein (BCA-P114) NPs. Rasouli and Tabrizian [94] combined oscillatory bubbles and sharp edges to fabricate PLGA NPs (Fig. 11d). Experiments were designed to assess each feature separately and in combination. It was shown that by combining the mixing features, mixing efficiency was considerably increased, even at high flow rates. A maximum mixing index of 85% (combination) at 20 μl/min was achieved, followed by 90% (bubbles) at 3 μl/min and 90% (sharp edges) at 1 μl/min. The mixing capability of the acoustic actuation of bubble-edge duo was compared to an MHF channel, where the former, outperformed in both size and PDI by generating PLGA NPs between 35 and 100 nm, over a range of flowrates (10–60 µL/min) and concentrations.

Similarly, Bachman et al. [241] also combined two mixing features in a PDMS microchannel. Here, however, sharp edges (active mixing) and tesla structures (passive mixing) were implemented to be able to operate at a wide range of flow rates. Separately, tesla structures are effective at mixing fluids at high flow rates, whereas sharp edges are effective at low flow rates. When the acoustic signal is “ON”, complete mixing occurs at all flowrates. However, when acoustic actuation is “OFF”, complete mixing only occurs at flow rates higher than 1500 µL/min. In combination, smaller PLGA NPs were synthesized (64.5–93.76 nm) in comparison to the tesla structures alone (75.56–177 nm).

**Fig. 12:**
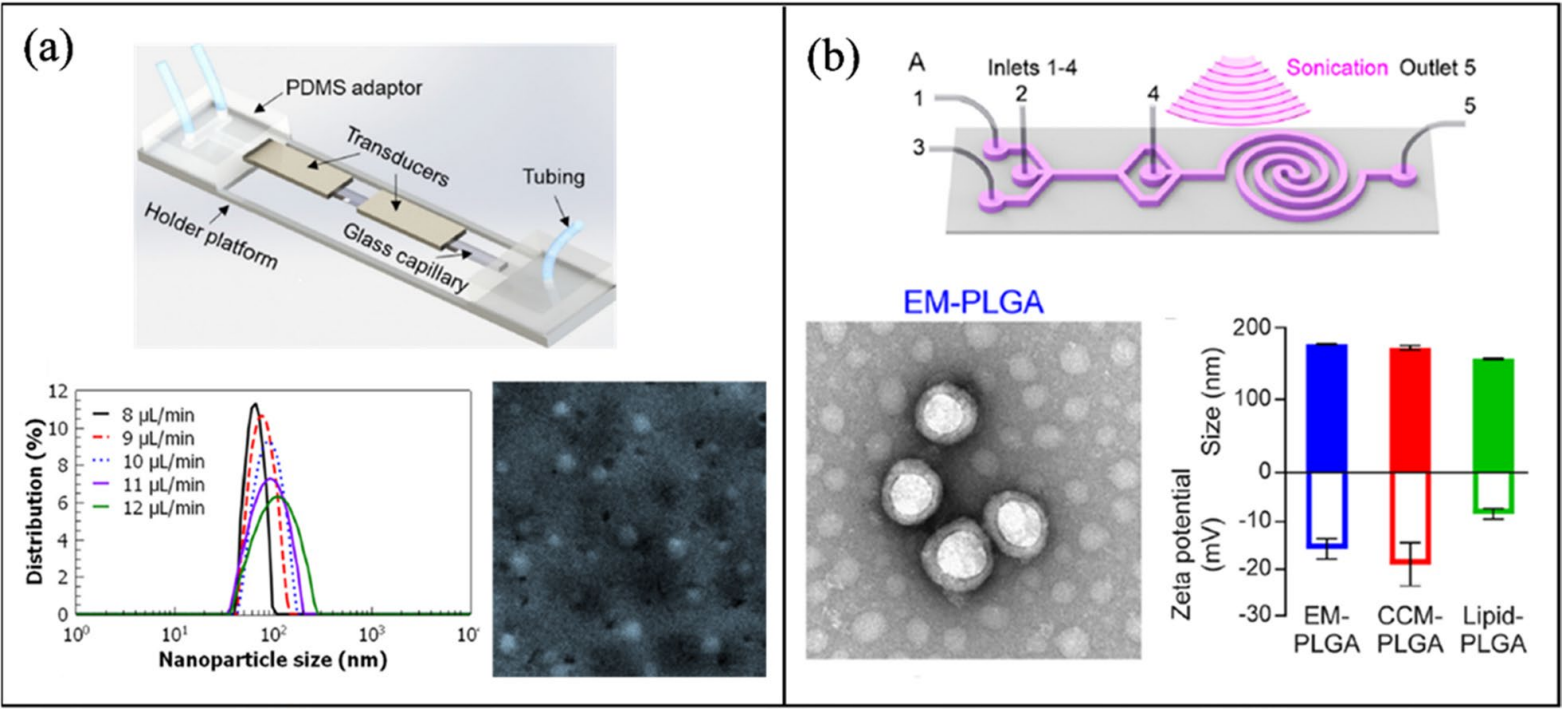
Acoustic micromixing for the fabrication of organic NPs (**a**) Flexural modes-acoustic streaming PLGA NPs synthesis (open access) [117]. **b** Hybrid lipid-PLGA NPs synthesis in a sonication-spiral microchannel (open access) [209]

Moreover, Hoogendijk et al. [242] tackled one of the main issues impeding large-scale production of PLGA NPs from microchannels: very low throughput. A three-stage micromixing platform was designed to produce Perfluorocarbon (PFC) encapsulated PLGA NPs. PFCs are hydrocarbon molecules where the hydrogen atoms are replaced with fluorine (or other halogens) atoms and are used in various applications, including imaging agents, and in the treatment of strokes and cancer [243]. Due to their chemical composition, PFCs exhibit hydrophobic and lipophobic properties, making them immiscible in both PLGA and aqueous solutions. As a result, mixing was performed in steps, two slit interdigital micromixers followed by an ultrasonic flow cell. Higher flow rates and sonication result in smaller and monodisperse particles with high encapsulation (60%). The platform was able to synthesize NPs with sizes ranging from 150 to 400 nm and 0.2 PDI by varying the flow rates (reaching a maximum flow rate of 40 mL/min) and changing the organic solvents (dichloromethane, chloroform, and ethyl acetate). Ozcelik and Aslan [117] demonstrated a low-cost and simple method of synthesizing PLGA NPs with glass capillaries and PDMS. To avoid the use of cleanroom facilities and the difficulties associated with it, Ozcelik and Aslan utilized commercially available rectangular glass capillaries with PDMS adapters for inlet and outlet ports. Piezoelectric transducers were glued to the capillaries to induce acoustic streaming through the excitation of different flexural modes. At 10 μL/min, the mixing time and efficiency were 103 ms and 85%, respectively. As the polymer to water flow rate ratio changed from 0.3 to 0.9, size increased from 65 to 100 nm, and PDI changed from 0.08 to 0.18. Where scanning electron microscopy (SEM) and DLS were used for hybrid NP morphological characterization, and size and PDI measurement respectively (Fig. 12a).

Liu et al. [209] proposed a passive/active mixing microfluidic platform for the development of a novel class of hybrid NPs. In this study, the fabrication of imaging agent loaded biomimetic NPs such as exosome membrane (EM) PLGA, cancer cell membrane (CCM) PLGA, and lipid coated (LC) PLGA were presented. The utilization of natural membranes provide an efficient way of reducing immune clearance and improving tumor-specific targeting [244]. The platform consisted of two stages, a straight channel followed by a spiral channel, with four inlets and one outlet. To prepare the NPs, EM (CCM, or lipid) in PBS was injected in the middle inlets while PLGA in organic solution was injected in the side inlets. The synthesis process was also done while submerged in an ultrasonic bath at 80 kHz where the ultrasonic waves exert an acoustic pressure (200 kPa) much higher than the critical compression stress of the natural membranes. This results in their rupture and reassembly around the PLGA core. Without sonication, the size and PDI of EM-PLGA NPs were 237.6 nm and 0.474, respectively, and decreased to 177.4 nm and 0.193 with sonication. In addition, sonication enhanced the membrane—PLGA coating process from 47.3 to 90.5%. Figure 12b shows TEM, NP size and ζ-potential measurements. Moreover, hybrid NP synthesis was also presented by Zhao et al. [245]. In this study lipid coated PLGA NPs were produced by a sequential process in a PDMS/sharp edges microchannel. Firstly, high molecular weight PLGA, (PLGA_70k_-PEG_2k_) in acetonitrile and water were injected separately into the microchannel and mixed by the acoustic streaming to form the PLGA core. Where high molecular weight PLGA can enhance the loading efficacy and release profile of drug carriers [246]. Secondly, a lipid (DSPE and cholesterol)/ethanol solution was injected into the channel forming shells on the PLGA cores by the sharp edges mixing.

##### Inorganic NPs

**Fig. 13:**
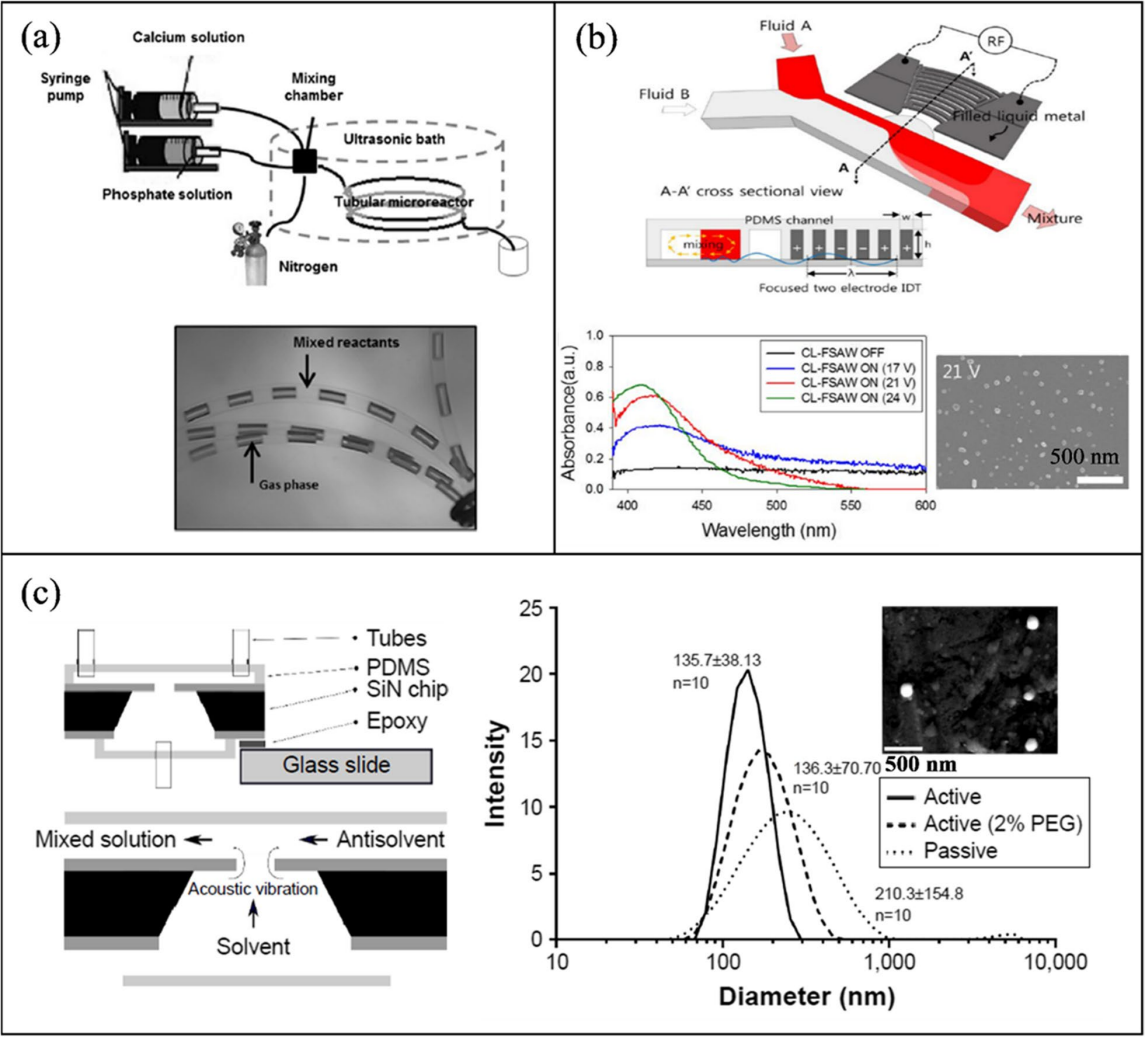
Acoustic micromixing for the fabrication of inorganic NPs (**a**) Hydroxyapatite NP synthesis in a tubular microreactor with sonication (open access) [210]. **b** SAW-acoustic streaming AgNP synthesis. Reproduced with permission from [248]. Copyrights © 2018 Elsevier. **c** Budesonide NPs production in microchannel with a vibrating membrane (open access) [250]

Castro et al. [210] presented the synthesis of hydroxyapatite (HAP) in a tubular microreactor with sonication (Fig. 13a). HAP is calcium-based mineral (Ca_5_(PO_4_)_3_(OH)) used in drug delivery applications due to its high biocompatibility [247]. The device consisted of two parts, initial mixing of the reactants (calcium hydroxide and orthophosphoric acid) in a mixing chamber followed by a tubular microreactor immersed in an ultrasonic bath operated at 40 kHz. NPs produced in this method were in the nanometer range, while NPs synthesized in the conventional stirring tank method were in the micrometer scale. Nam et al. [248] evaluated the use of conductive liquid (eutectic gallium indium) instead of patterned metal IDT for traveling surface acoustic wave (TSAW) silver nanoparticle (AgNP) synthesis. The device consisted of a 128° Y-cut lithium niobate (LiNbO_3_) substrate and PDMS, where the PDMS contains the main microchannel along with the IDT (9.2 MHz resonance frequency) cavity for the conductive liquid to be injected. This setup provides two main advantages: (i) precise control of IDT and microchannel positioning, and (ii) elimination of the metal deposition step. Without TSAW, AgNPs were unstable, aggregated, and showed a large size distribution. As the voltage was increased above 13 V, 20–90 nm NPs were formed and complete mixing was achieved at 80 μL/min. Size and size distribution, and morphology were characterized by UV–Vis spectroscopy and field emission scanning electron microscopy (FE-SEM) (Fig. 13b). Similarly, Liu et al. [249] designed two experiments using surface acoustic waves (SAW) to synthesize gold nanoparticles (AuNP). Two SAW IDT layouts were proposed: orthogonal and rotary. The orthogonal design had four IDTs across from each other around a circular chamber, while the rotary design had the IDTs shifted to form a vortex in the chamber. Overall, SAW suppresses agglomeration and deposition, however, at high voltages, the orthogonal design traps and aggregates AuNPs at nodes due to the formation of standing waves. The rotary design produced AuNPs with good monodispersity and small size.

Le et al. [250] presented a microchannel with a vibrating membrane (Fig. 13c) to synthesize an asthma medication known as Budesonide. It is one of the most important medications for pulmonary diseases, yet its low solubility limits its efficacy in the body [251]. The device consisted of a silicon base with a square cavity and thin edges, a PDMS channel, and a piezoelectric transducer. Solvents flow from the bottom through the square section in to the PDMS channel. As the transducer is actuated (177.6 kHz), the edges vibrate to form strong vortices that completely mix the fluids. Budesonide NPs were synthesized with an average diameter of 135.7 nm and a PDI of 0.044 (3 ms mixing time) in comparison to 210 nm and a PDI of 0.238 without actuation (SEM and DLS characterization). In another work by the same group [252], budesonide NPs were synthesized in a similar platform; however, the cavity had a star cross section and the NPs had an average size of 80 ± 22 nm and a mixing time of 4.1 ms.

#### Electrical/thermal NP synthesis

**Fig. 14:**
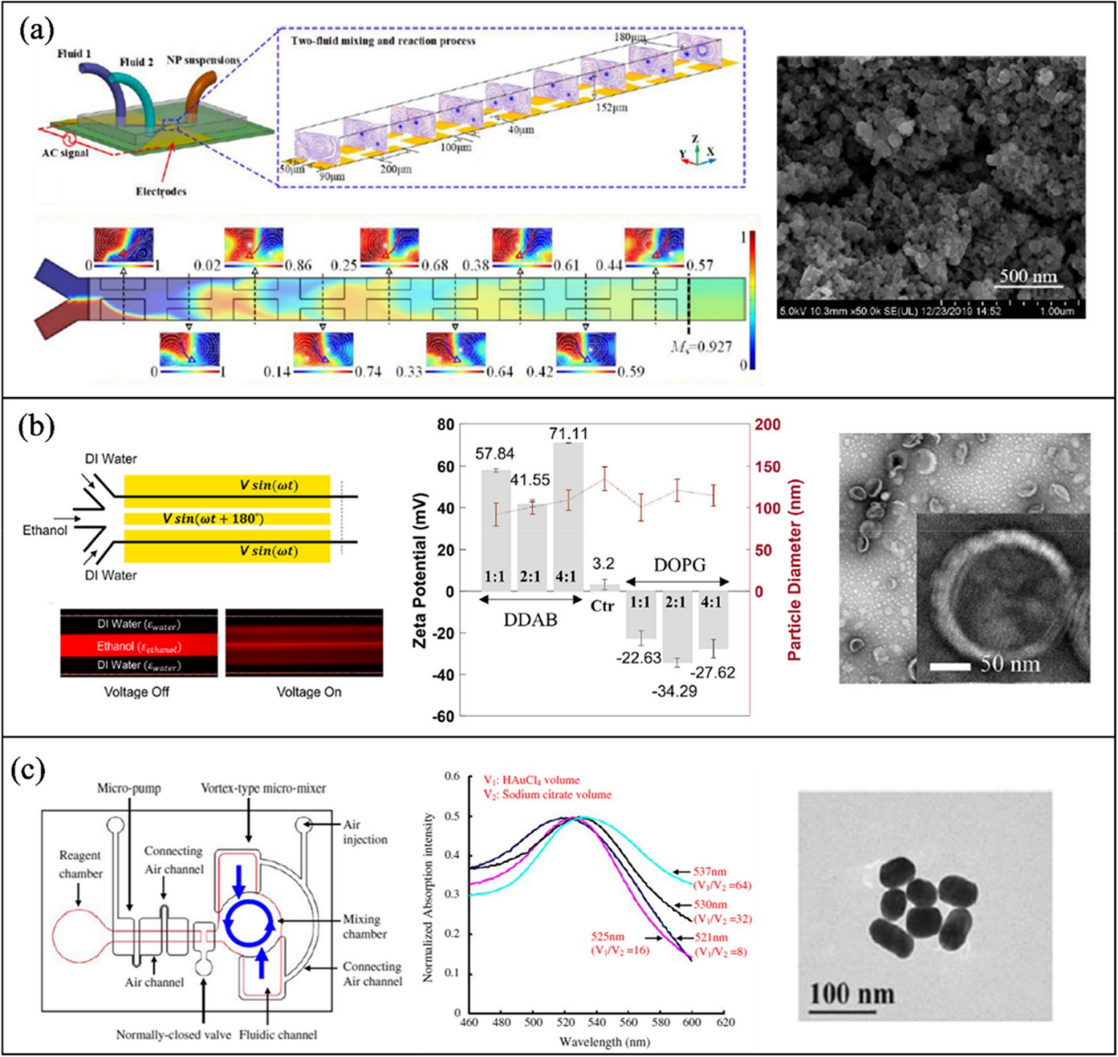
Active mixing methods for the synthesis of NPs (**a**) Alternating current electrothermal with staggered sequential electrodes for the synthesis of Prussian NPs (open access) [203]. **b** Electrohydrodynamics mixing with three parallel electrodes to synthesize cationic, anionic, and neutral liposomes (open access) [234]. **c** AuNPs synthesis in a pneumatic micromixer (open access) [256]

Zhang et al. [253] utilized an indium tin oxide (ITO) microheater to synthesize copper (I) oxide (Cu_2_O) NPs. This metal oxide has been suggested for biomedical applications because of its antimicrobial properties [254]. The micromixer was composed of a PDMS channel on top of an ITO film and a glass substrate. Where the ITO film is deposited asymmetrically along the length of the channel, such that when a DC current is applied, fluids are unevenly heated, resulting in the formation of a thermal buoyancy convection current. At an applied voltage of 8 V and a flow rate of 300 nL/s, minimum NP size was achieved at 680 ± 9 nm and decreased to 477 nm with precursor dilution (8:1). Conversely, Sun et al. [202], used vortices induced by alternating current electrothermal (ACET) flow to synthesize and guide barium sulfate and Prussian blue (Iron(III) ferrocyanide) NPs (diagnostic agents [255]). The microchannel consisted of PDMS, glass, two pairs of ITO electrodes along the length of the channel, and a central floating electrode. Optimum conditions were investigated in terms of voltage (*V*_1_ = 12 V, *V*_2_ = 20 V), frequency (1 MHz), and phase difference (180°). As a result, spherical barium sulfate NPs were produced with a diameter ranging from 75 ± 17.5 nm to 709.9 ± 94.2 nm depending on different additives. In addition, Prussian blue NPs were synthesized and guided into the lower outlet. In another work by the same group and using the same mixing approach [203], 100 nm cobalt-iron prussian NPs were synthesized with 9 pairs of staggered sequential electrodes (Fig. 14a).

Modarres and Tabrizian [234] demonstrated a novel approach using electrohydrodynamics to synthesize cationic, anionic, and neutral liposomes. The electric field was applied to the PDMS channel by a pair of straight gold electrodes along the channel. Water and lipid/ethanol were the two solvents utilized, and as a result, an electrical body force was generated due to the difference in electrical properties between them. At 1 mg/mL, 10:1 FRR, and 50 μL/min, liposomes (DPPC and cholesterol) had a size of 126.7 ± 0.7 nm. Cationic and anionic liposomes were synthesized by the addition of DDAB and DOPG, respectively, to the DPPC and cholesterol. Highly positive liposomes were synthesized between 91.8 and 109.1 nm, whereas anionic liposomes were produced between 100.4 and 114.6 nm. NP characterization was determined by DLS, TEM, and ζ-potential measurements (Fig. 14b). The same mixing mechanism was applied in ref. [95], however, wavy sinusoidal electrodes were used to synthesize DPPC liposomes.

#### Other methods

Yang et al. [256] developed a pneumatic micromixer for AuNP synthesis. The micromixer has two PDMS layers (prepared from PMMA molds), a fluidic channel and an air channel (Fig. 14c). The mixing chamber walls are connected to air channels, where the supplied pressurized air operating at 6 Hz can deflect the walls of the chamber, creating vortices and achieving complete mixing within 0.7 s. By varying the volume of the reactants, they were able to synthesize AuNPs ranging from 19 to 58 nm (measured by UV–Vis spectroscopy and TEM). Similarly, Xin et al. [124] utilized a pneumatic mechanism to synthesize cadmium sulfide (CdS) quantum dots. The device consisted of a glass base and two PDMS layers, where the middle PDMS layer contains an “S” shaped mixing chamber. The last PDMS layer contains a thin diaphragm outlining the mixing chamber. The diaphragm vibrates and induces disturbances in the fluid when the compressed nitrogen gas is pumped. CdS quantum dots are produced by mixing sodium polyphosphate and Cd(NO_3_)_2_·4H_2_O. Pneumatic micromixing resulted in smaller and more uniform quantum dots in comparison to the conventional co-precipitation method under magnetic stirring. Other researchers utilized piezoelectric transducers as micropumps to synthesize AuNPs by alternating the pumping between the two inlets. Sugano et al. [204] demonstrated valveless microfluidic pulsed mixing by using two piezoelectric transducers (for each inlet) as micropumps for the synthesis of AuNPs. Here, two solutions are alternatively pumped at a specific frequency into the channel to produce a pulsed layer of each solution to increase the contact area. Increasing the switching frequency to 200 Hz resulted in the best mixing time of 95 ms and a size of 42.7 nm.

Verma and Kumran [257] fabricated a split-inlet PDMS microchannel with hard and soft sections to mix fluids by the induced dynamical instability. The dynamic coupling between the fluid and the walls results in flow instability and mixing similar to turbulent flows. The transition to turbulent-like flows depends on the shear modulus of the walls. Where the soft PDMS section has a lower shear modulus than the hard PDMS. For example, standard protocol PDMS has a shear modulus of 0.55 MPa, while the soft section has a shear modulus of 18 kPa with a transition Reynolds (Re) number of 200. Small (< 5 nm) and uniform AuNPs were produced at Re greater than the transition critical number.

Lie et al. [258], proposed an alternative PMMA microfluidic device with piezoelectric actuation. The device has a circular chamber with four piezoelectric elements distributed evenly around the chamber and connected to the inlets. The transducers induce rotation, uniform mixing, and suppress agglomeration. As the reagents enter the chamber, they are continuously pulled in and discharged by the transducers (two positioned opposite from each other at a time), which results in vortices and self-circulating flow. The proposed platform produced small particles (10.31 ± 2.38 nm) with good size distribution and no agglomeration at 80 V and 3.83 kHz.

Xia et al. [132] synthesized SLN using a microfluidic mixer which contains an oscillator mixer with an elastic diaphragm. Such that above a certain critical pressure, the diaphragm spontaneously vibrates, transforming the laminar flow to oscillatory flows. A pressure of 4.5 bar was determined to produce the optimal mixing and synthesized particles with an average diameter of 93.4 nm and 0.184 PDI.

## Computational approaches to study NPs

**Fig. 15:**
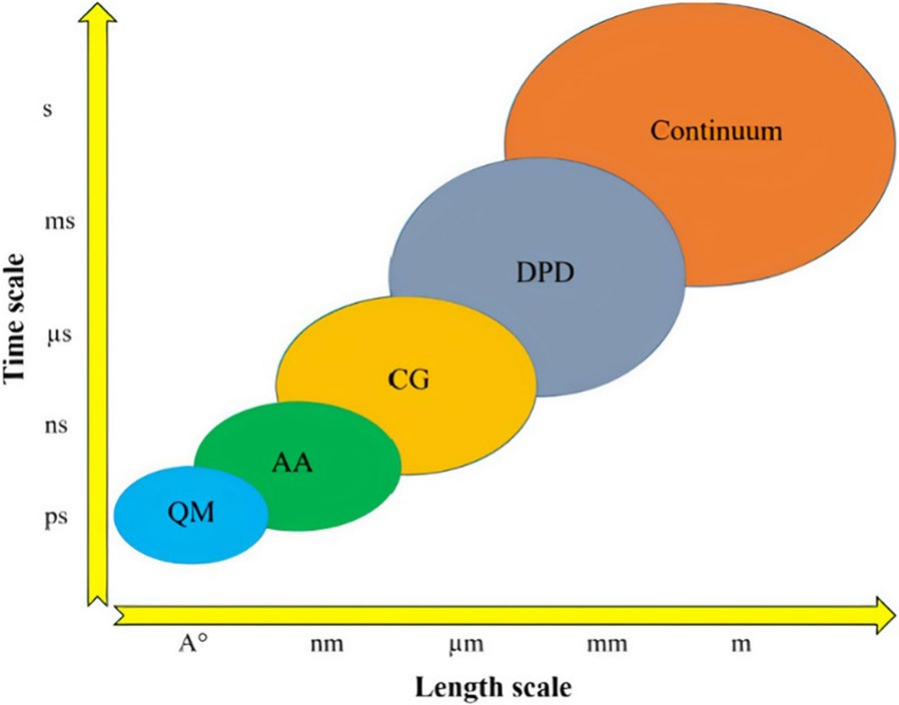
Classifications of the simulation methods based on the length and time scales. QM: Quantum Mechanics; AA: All-Atomistic; CG: Coarse-grained; DPD: Dissipative Particle Dynamics

Despite many advances in design, synthesis, and dynamics of nanoparticles and their interactions with other nanoparticles/cells and extracellular medium, there is still a dearth of knowledge about the field. This is because of the innumerable inherent and practical restrictions faced by researchers during the experiments to capture the complete structural and functional resolutions for nanoparticle-based systems. Any systematic approach to varying the properties of nanoparticles, e.g., size, morphology, or surface charge, can be extortionately time, labor, and cost intensive. Consequently, relying solely on experiments to predict the workings of a nano-system is a challenging task. To address this shortcoming, theoretical techniques comprising both analytical as well as computational methods can prove handy in generating quick results without performing time-consuming and complicated experiments. Furthermore, these techniques also allow the prediction of appropriate conditions for further investigations and, hence, are an important tool to complement experiments and provide invaluable assistance in designing novel formulations with enhanced effectiveness. Based on the spatio-temporal scales (Fig. 15), which range from femtosecond to millisecond in time and Angstrom to millimeter in length, the computational techniques can be broadly placed into four categories: Quantum Mechanics (QM) calculations, atomistic Molecular Dynamics (AMD) simulations, coarse-grained (CG) molecular simulations, and continuum mechanics. The most accurate among these methods are the QM calculations, but their scope is limited, whereas the continuum mechanics simulations miss the molecular details. Hence, the main focus of the current section is to cover the molecular simulations for the nanoparticles. Nevertheless, a brief account of each simulation method is described below. Furthermore, the salient features of each method are summarized in Table 4.

**Table 4:** Most commonly employed simulation techniques for nanoparticles

Simulation type	Representatives	Force field	Time scale	Spatial-scale	Advantages	Disadvantages	Applications
Quantum mechanical (QM) Calculations	Density functional theory (DFT) [261]		10^–15^ s	$10^{3} { }$ atoms	Most accurate and detailed molecular method	Limited spatio-temporal scales	Structure, stability, and electronic properties of a nanomaterial
	QM/molecular mechanical(MM) method [262, 263]						
All-atom molecular simulation	Atomistic Monte Carlo (MC) [264]		10^–9^ s	1–10 nm	The atoms are explicitly modeled Parameters are obtained based on experimental and QM calculations	Limited spatio-temporal scales Lack of suitable parameters for NPs	Adsorption, etc. Compatibility studies, molecular diffusion, interface chemistry, etc
	Atomistic molecular dynamics (AMD) simulation [265]	GROMOS [266]; CHARMM [267]; OPLS [268]; AMBER [269]					
Coarse-grained molecular simulation	Coarse-grained (CG) molecular dynamics (MD)	MARTINI [270]; L–J [271]	10^–9^–10^–6^ s	10–100 nm	Increase of spatio-temporal scales upto 2 orders $O\left( 2 \right)$ of magnitudes as compared to AMD	Does not provide atomic level resolution Implementation is not straight-forward Beads can cross -over each other	Membrane Fusion Processes [272], Phase separation, self-assembled structure, cell membrane, etc
	Dissipative particle dynamics (DPD) [273]	Soft-Potentials					

### Quantum mechanics (QM) calculations

Among all molecular modeling methods, Quantum Mechanics (QM) calculations provide the most accurate and most detailed simulations. QM calculations are based on solving Schrödinger’s wave equation [259] and are widely used to model chemical reactions, the creation/breakage of chemical bonds, and the electronic structure of materials. The QM methods are concerned with modeling the nuclei and electron states.

When the length scale of interest in the problem is in the order of the de Broglie thermal wavelength, quantum effects become relevant. The following formula yields the de Broglie wavelength scale [260].

3 $$\lambda = \sqrt {\frac{{h^{2} }}{{2\pi mk_{b} T}}}$$

where $\lambda$ is the de Broglie wavelength, *h* is Planck’s constant, m is the mass, *k*_*B*_ is Boltzmann constant, and *T* is the temperature. When examining biological molecules suspended in water, the mass of these molecules is significantly more than the mass of water, implying that their thermal wavelength is substantially less than that of water, according to the de Broglie wavelength formula. Therefore, the quantum effect occurs in water molecules before it manifests in living species. As a result, in order to test the quantum effect, one must first compute it for water molecules. According to the previous formula and taking into account the mass of one mole of water (18.01 × 10^–3^ kg), the mass of a single water molecule is roughly 2.99 × 10^–26^ kg, and the de Broglie wavelength is around 2.4 × 10^–11^ m. (i.e., 0.024 nm). Given that the average intermolecular distance between water molecules is roughly 0.3 nm, the de Broglie wavelength is more than ten times smaller than the average intermolecular distance between water molecules, and hence the quantum effects will be insignificant in water. As a result, the de Broglie wavelength for biological species (like liposomes) flowing in plasma or water will be significantly lower than that of water, and no quantum effects will be observed.

An important attribute of QM applications is that they do not require any prior knowledge of the experimental or empirical data. However, these calculations are still limited to modeling the response of systems containing atoms up to the order of $10^{3}$ atoms for time up to $10^{ - 15}$ seconds. Consequently, they are only used to simulate the properties of quantum dots (QDs), carbon nanotubes, and systems that involve similar length and time scales. For nano-systems involving larger entities such as liposomes, the QM calculations are currently of limited use and hence are not preferred.

### Atomistic molecular dynamics (AMD)

In AMD simulations, the unit block is an individual atom, and each atom is modeled explicitly. As a result, the AMD simulations have the capability to describe material attributes down to atomic-level resolution. The classical atomistic simulation methods do not model the electrons explicitly. Instead, their effects are averaged out and are included in the simulations via the interatomic potential, $E\left( {\vec{r}_{1} , \vec{r}_{2} , \vec{r}_{3} \ldots ,\vec{r}_{N} } \right)$, in which a system comprising of $N$ atoms and having the potential energy $E$ is a function of the position vectors $\vec{r}_{i}$ of the atoms. The most well-known mathematical approximation used in AMD simulations is the Born–Oppenheimer approximation [274] in which the wave functions of electrons are assumed to be separable from those of the nuclei in molecules. The electrons are assumed to respond to the changes in atomic locations much quicker compared to the atomic nuclei because the mass of an electron is much smaller than the mass of a nucleus. As a result, the atomic positions determine the potential energy of a system of interacting atoms. The interatomic potentials describing the approximate interaction between the atoms are mostly defined by analytic functions that are parameterized by data-fitting to the existing experimental results. Direct QM–based computations utilized in the first principle (ab initio) methods may also be used to analyze interatomic potentials. However, the first principle methods are compute-intensive and effective in much smaller scaled systems (i.e., up to a few thousand atoms). To make significant advances in the field and increase the ambit of AMD simulations to perform accurate quantitative investigations about the characteristics and behavior of nanostructured materials, it is imperative to introduce accurate and simple interatomic potential functions that are easier to compute mathematically. The following are some of the most prevalent atomistic computational approaches used in materials research.

#### Monte carlo (MC) methods

The MC method, most generally, includes a broad range of stochastic methods that generate a series of new states for the system under investigation without taking into account the Newton’s equations of motion. Because the MC approach does not employ equations of motion, it does not involve the idea of explicit time and can therefore only be used to simulate events in thermodynamic equilibrium. As a result, the MC approach is not an appropriate choice when dealing with a system’s dynamic properties, as these are time-dependent attributes. It is worth mentioning that the MC method is not meant for atomistic simulations alone; it can be utilized to simulate any scale provided an accurate probabilistic mode is available. The MC method follows a Markov chain process to compute a system’s new state from the current one. Initially, the original state of particles in the system is defined. A Monte Carlo move then modifies the original states of the particles, which are either approved or disapproved based on the “acceptance condition” that satisfies the balance conditions so that the equilibrium can be described properly. After this step, the required property of the system is computed, and the moves are repeated several times to obtain the average of the required property of interest. In this way, the thermodynamic properties of the system at equilibrium are computed using principles based on statistical mechanics. Another important aspect is that the MC method should use an ergodic scheme [275, 276].

The three major steps in the MC method are: (i) converting the physical incidence into a suitable probabilistic model; (ii) computing the solutions of the utilized model via*.* stochastic sampling “computer experiments”. (iii) data processing and analysis by employing statistical means. In the sampling step, the algorithm utilizing either a single or weighted sampling technique is utilized. The simple sampling employs a uniform distribution of random numbers, whereas weighted sampling generates numbers using a distribution tailored to the specific problem at hand. The weighted sampling algorithm is the underlying principle of the Metropolis MC method [277], which is based on the weighted sampling technique. It should be emphasized that although the original MC approaches were inherently intended to investigate a system’s equilibrium states, the integration of Ising lattice model with the Potts-type MC models facilitated the expansion of MC methods in modeling the evolution of microstructures [278]. This category of MC models is sometimes called kinetic Monte Carlo (KMC) models [279–281] since they are based on internal kinetic measure (e.g., number of MC steps).

For an ensemble of $N$ atoms, the Metropolis MC achieves a new configuration of atoms, selecting an atom and displacing it from its starting location $i$ to a trial position $j$ temporarily. This corresponds to a change in the phase space of the system from an initial state ${\Gamma }_{i}$ to the trial state ${\Gamma }_{j}$, As a result, the system’s Hamiltonian changes from ${\text{H}}({\Gamma }_{i} )$ to ${\text{H}}({\Gamma }_{j} )$ depending on the specific interactions that the model is taking into account. This change in system’s Hamiltonian ${\Delta H}({\Gamma }_{i \to j} )$ can be represented as:

4 $${\Delta H}({\Gamma }_{i \to j} ) = H\left( {{\Gamma }_{j} } \right) - H\left( {{\Gamma }_{i} } \right)$$

The enforced displacement of the atom and new configuration of atoms is accepted if the enforced change lowers the system’s energy level, i.e., ${\Delta H}({\Gamma }_{i \to j} ) < 0$. In case the system’s energy is not lowered, the enforced displacement is only approved with a probability $P_{i \to j}$ which can be expressed as:

5 $$P_{i \to j} \propto \exp \left( { - \frac{{{\Delta }H\left( {{\Gamma }_{i \to j} } \right)}}{{k_{B} T}}} \right)$$

where $k_{B}$ represents the Boltzmann’s constant, and $T$ is the system’s temperature. To test the changed configuration of atoms, a random number with a range from 0 to 1 is generated in the Metropolis MC. The enforced displacement is approved only if that random number $\le \exp \left( { - \frac{{{\Delta }H\left( {{\Gamma }_{i \to j} } \right)}}{{k_{B} T}}} \right)$. If the displacement is not approved, the original location is used as the new position, and the process is then repeated with a different randomly selected atom. For nanomaterials, the technique is mostly applicable to studying the equilibrium shapes of individual nanostructure constituents (such as nanoparticles), and surface composition or structure [277]. Kinetic MC methods can be effectively employed if the changes in nanomaterial’s structure/composition are determined by a relatively limited number of thermally triggered elementary processes, such as the evolution of shapes of small crystallites because of surface diffusion [282], or the formation of 2-D fractal-dendritic islands [283].

#### Molecular dynamics

Molecular dynamics (MD) is a simulation technique that computes and describes the time-evolution of a system by employing Newton’s equation of motion. For a system of $N$ particles (in case of AMD methods, particles refer to the atoms), the motion of the particles is described as:

6 $$\frac{{m_{i} {\text{d}}^{2} \vec{r}_{i} }}{{{\text{d}}t^{2} }} = \vec{F}_{i} ,\,\,\,i = 1,2, \ldots , N$$

where $m_{i}$ and $\vec{r}_{i}$ represent the mass and position vector of $i\,{\text{th}}$ particle, respectively; and $\vec{F}_{i}$ represents the force that acts on the $i\,{\text{th}}$ particle as a result of its interaction with the other particles in the system. This force $\vec{F}_{i}$ is computed by the gradient of the $E\left( {\vec{r}_{1} , \vec{r}_{2} , \vec{r}_{3} \ldots .,\vec{r}_{N} } \right)$, which is the inter-particle interaction potential and depends on the position vectors of the particles in the system. Mathematically, it is stated as:

7 $$\vec{F}_{i} = - \vec{\nabla }E\left( {\vec{r}_{1} , \vec{r}_{2} , \vec{r}_{3} \ldots .,\vec{r}_{N} } \right)$$

The MD simulation starts with initializing the positions, velocities, and the nature of the inter-particle interaction potential for all the particles comprising the system. After the initialization step, Eq. (6) is solved iteratively to provide the time evolution of the positions $\left( {\vec{r}_{i} } \right)$, and velocities $(\vec{v}_{i}$) of all the particles. These trajectories are then used to compute other derived quantities, such as the spatio-temporal evolution of thermodynamic and structural attributes of the system. The most popular integration method is the Verlet scheme. By using the Taylor expansion, it considers the positions at a particular time step $\left( t \right)$ and the previous time step $\left( {t - {\Delta }t} \right)$, along with the accelerations at time $\left( t \right)$, to calculate the new positions at the next time step $(t + {\Delta }t$). If ${\varvec{r}}_{{\varvec{i}}} \left( t \right)$ represents the positions at the current time step, ${\varvec{r}}_{i} \left( {t - {\Delta }t} \right)$ the positions at the previous time step, and ${\varvec{a}}_{{\varvec{i}}} \left( t \right)$ the acceleration at time step $\left( t \right)$. The new position ${\varvec{r}}_{i} \left( {t + {\Delta }t} \right)$ at the next time $\left( {t + {\Delta }t} \right)$ can be stated as:

8 $${\varvec{r}}_{i} \left( {t + \Delta t} \right) \approx 2{\varvec{r}}_{i} \left( t \right) - {\varvec{r}}_{i} \left( {t - {\Delta }t} \right) + a_{i} \left( t \right)\left( {\Delta t} \right)^{2}$$

The velocities $v_{i} \left( t \right)$ and $v_{i} \left( {t + \frac{1}{2}{\Delta }t} \right)$ at times t and $t + \frac{1}{2}{\Delta }t$ can be estimated as:

9 $${\varvec{v}}_{i} \left( t \right) \approx \frac{{{\varvec{r}}_{i} \left( {t + {\Delta }t} \right) - {\varvec{r}}_{i} \left( {t - {\Delta }t} \right)}}{{2{\Delta }t}}$$

10 $${\varvec{v}}_{i} \left( {t + \frac{1}{2}{\Delta }t} \right) \approx \frac{{{\varvec{r}}_{i} \left( {t + {\Delta }t} \right) - {\varvec{r}}_{i} \left( t \right)}}{{{\Delta }t}}$$

The fundamental advantage of MD is that it only requires knowledge of how the particles (atoms) interact with each other, i.e., inter-atomic interactions. It does not require any assumption regarding the nature of the processes under investigation. This makes the MD method an attractive approach to conduct the so-called “computer experiments” and discover new, interesting physical phenomena. Thus, the MD method is influential in assisting the real experiments. As a matter of fact, in some instances, the performance of the MD surpasses that of the real experiments. For instance, the investigation of some fast non-equilibrium methods is often not possible in real-world experiments. As a result, the complete information of such processes is difficult to capture in real experiments. The MD method, on the contrary, can easily handle such problems, thus helping the researchers to understand the unexplored phenomena of interest.

The MD simulations typically compute solely the interactions between pairs of particles (or atoms). Hence, to approximate force fields, two-body potentials are used, and the 2-dimensional force matrix of pairwise interactions is used to characterize the system's energy. The force field typically comprises of bonded terms (representing the interactions between covalent-bonded atoms), and non-bonded terms (that capture the interactions for the atoms that are not connected with covalent bonds). The bonded interactions include bond stretch ($E_{{{\text{bond}}}}$), angle bend ($E_{{{\text{angle}}}}$), dihedral angle ($E_{{{\text{dihedral}}}}$), and improper angle ($E_{{{\text{improper}}}}$) interactions. The non-bonded interactions approximate the van der Waals (vdW) interaction ($E_{{{\text{vdw}}}}$) that are modeled by utilizing a Lennard–Jones (L–J) potential. For charged entities, the electrostatic interactions ($E_{{{\text{elec}}}}$) is introduced. The mathematical details of these models are given below:

11 $$E_{{{\text{vdw}}}} = 4\epsilon \left[ {\left( {\frac{\sigma }{r}} \right)^{12} - \left( {\frac{\sigma }{r}} \right)^{6} } \right]$$

12 $$E_{{{\text{elec}}}} = \frac{{q_{i} q_{j} }}{{4\pi \epsilon_{0} r_{ij} }}$$

13 $$E_{{{\text{improper}}}} = k_{\varphi } \left( {\varphi - \varphi_{0} } \right)^{2}$$

14 $$E_{{{\text{dihedral}}}} = k_{d} \left[ {1 + \cos \left( {\eta \zeta - \delta } \right)} \right]$$

15 $$E_{{{\text{angle}}}} = k_{\theta } \left( {\theta - \theta_{0} } \right)^{2}$$

16 $$E_{{{\text{bond}}}} = \frac{{k_{b} }}{2}\left( {l - l_{0} } \right)^{2}$$

17 $$E = E_{{{\text{vdw}}}} + E_{{{\text{elec}}}} + E_{{{\text{improper}}}} + E_{{{\text{dihedral}}}} + E_{{{\text{angle}}}} + E_{{{\text{bond}}}}$$

In these equations, the parameters such as $k_{\varphi }$, $k_{d}$, $k_{\theta }$, $k_{b}$, and $\theta$ typically depict empirical parameters to match physical properties obtained via experiments or quantum mechanical (QM) calculations. Common force fields in the MD simulations are GROMOS [266], AMBER [269], CHARMM [267], and OPLS [268]. The associated software packages are mostly developed with the same names, such as GROMOS [266], AMBER [269], and CHARMM [267]. Nevertheless, other general-purpose programs have also been developed to perform the MD, MC, QM and even coarse-grained calculations. Some examples include BOSS [284], Abalone [285], Desmond [286], Discovery Studio [287], and LAMMPS [288].

### Coarse-grained (CG) molecular dynamics

**Fig. 16:**
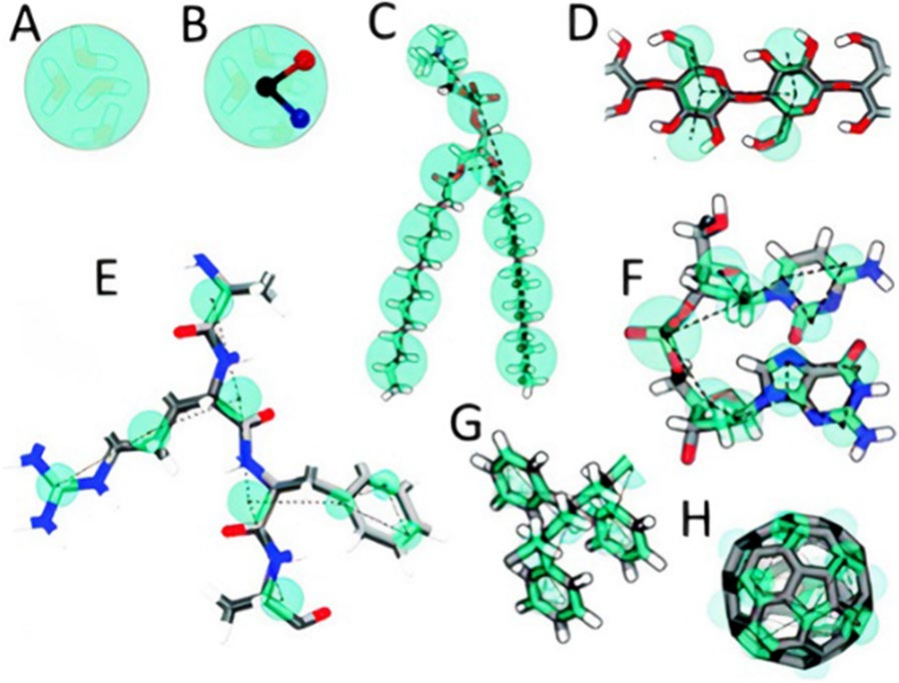
Coarse-grain mapping strategy using the Martini model for (**A**) standard water bead with 04 water molecules, (**B**) polarizable water molecule with charges embedded in it, (**C**) DMPC lipid, (**D**) Polysaccharide fragment, (**E**) Peptide, (**F**) DNA Fragment, (**G**) Polystyrene Fragment, (**H**) Fullerene molecule. Reproduced with permission from ref. [291]. Copyrights 2013 Royal Society of Chemistry

Despite the widespread popularity of AMD simulations in investigating physical phenomena, they are limited to the atomistic length scales and fail to capture several exciting phenomena that happen on length scales greater than the atomistic level. To address these spatio-temporal limitations of AMD, the concept of “coarse graining” was introduced, in which several atoms/molecules are combined together to represent a single particle (often referred to as a bead). As a result, the degrees of freedom of the system being simulated are reduced significantly [289]. However, because of the smooth interactions of these coarse-grained particles, a much larger time step (i.e., 10^1^ fs) is possible in CG-based MD (abbreviated as CG MD) simulations compared to the AMD simulations (where the typical time step is 10^0^ fs). A well-known and most common force field utilized in CG MD is the Martini force field [270] introduced by Marrink et al. [290]. Although the Martini FF was originally intended to model lipids, its applicability was later extended to model a variety of other biological molecules, such as proteins, peptides, polysaccharide, cholesterol, fullerene, and even DNA fragments. The Martini FF model is built on a four-to-one mapping technique, which means four heavy atoms are used to represent a CG bead. This choice of mapping provides an optimized balance between the computational costs and the chemical representation of the underlying structure. For example, in the case of water, the Martini model maps four real water molecules onto a single CG water bead. The Martini model also models the ions, along with their first hydration cell, as a CG bead. Depending on the chemical nature of a structure, a Martini particle can be classified either as non-polar (N), polar (P), apolar (I), or charged (Q). The subtypes within above-mentioned four major types are identified by either a letter ([d] = donor, [a] = acceptor, [da] = both, [0] = none) signifying the hydrogen-bonding capabilities type, or by a number (from [1] = low-polarity to [5] = high-polarity) depicting the degree of polarity. As a result, a total of eighteen types serving as building blocks are available in the Martini mapping model. Figure 16 represents the mapping model for water and a variety of molecules.

Similar to the classical FFs in AMD methods, the Martini model also describes the interactions of Martini beads in bonded and non-bonded terms. The bonded interactions are the same pairwise potential energy functions as those utilized in classical AMD simulations. Similarly, the non-bonded interactions are also characterized by the L-J and columbic (for charged entities) potentials. It should be noted that the Martini model uses shifted forms of these (i.e., L–J and Columbic) potentials so that the potential reduces to zero in a smooth manner as the inter-bead distance between the interacting beads increases the cut-off radius $r_{cut - off}$. The optimization of parameters in both bonded and non-bonded interaction models is based on experimental data and AMD simulations [270]. In general, two types of CG models with respect to the solvent are used: explicit solvent and implicit-solvent CG models. In explicit-solvent CG models, the particles are introduced as two-dimensional membranes that are immersed in a solvent that occupies the bulk of the simulation box. These models explicitly take into consideration the hydrophobic interactions between the solvent and the target particles (such as lipids) in order to keep the membrane stable in the fluid phase.

However, mostly ~ 90% of the computational time is spent on computing the solvent’s equations of motion. Consequently, the spatial–temporal scales of the explicit-solvent CG models are limited. Therefore, to decrease the computational load, implicit-solvent CG models (also known as solvent-free models) were introduced, which involve a much smaller number of particles compared to the former models. Here, the interactions (either hydrophobic or hydrophilic) between solutes and solvents are represented by the intermolecular interactions. However, for the simulations involving water as a solvent, accurate representation of the hydrophobic interactions to develop solvent-free models is not a straightforward task.

### Dissipative particle dynamics (DPD)

The DPD method is also a coarse-grained simulation method that operates on mesoscopic spatial–temporal scales and can accurately model the hydrodynamic interactions among a variety of species. DPD can be regarded as a coarse-grained version of the MD technique. The basic unit of a DPD simulation is a DPD particle (sometimes referred to as a DPD bead), which in fact, is a representation of a large number of solute/solvent molecules. Although it was originally designed to model the hydrodynamic behavior of complex fluids, its applicability has been enhanced over the years, and it is successfully applied to a variety of other phenomena, such as investigating the lipid bilayers at the mesoscales. In a DPD simulation comprising of “$N$” DPD beads, a soft pairwise-interaction between these beads is used in contrast to the hard-core interactions between actual molecules in MD simulations. Thus, a much larger time and length scale can be simulated using this method. Again, the time evolution of the DPD beads is governed by Newton’s law of motion. For the two interacting beads ($i$ and $j$), the ${\varvec{f}}_{i}^{DPD}$ comprises three different kinds of pair-wise additive forces, which are a function of the relative spacing and the velocities of the interacting DPD beads. The three forces are: (i) conservative force $({\varvec{F}}_{ij}^{C} )$, which is a soft repulsive force and represents the compressibility of the fluid, (ii) dissipative force $({\varvec{F}}_{ij}^{D} )$, which represents the viscosity of the fluid and tends to cool down the system, and (iii) random force $({\varvec{F}}_{ij}^{R} )$, representing the thermal energy of the DPD system and heating it up.

18 $${\varvec{f}}_{i}^{DPD} = \mathop \sum \limits_{j \ne i} {\varvec{F}}_{ij}^{DPD} = \mathop \sum \limits_{j \ne i} {\varvec{F}}_{ij}^{C} + {\varvec{F}}_{ij}^{D} + {\varvec{F}}_{ij}^{R} .$$

The conservative force (${\varvec{F}}_{ij}^{C} )$ is given by the relation:

19 $${\varvec{F}}_{ij}^{C} = \left\{ {\begin{array}{*{20}l} {a_{ij} \left( {1 - \frac{{r_{ij} }}{{r_{c} }}} \right)\hat{\user2{r}}_{ij} , } \hfill & { (r_{ij} < r_{c} ).} \hfill \\ {0,} \hfill & {(r_{ij} \ge r_{c} ).} \hfill \\ \end{array} } \right.$$

where $r_{c}$ is the unit of length-scale called the cut-off radius and represents the sphere of influence for the interactions of DPD beads. That is, the DPD beads only interact if the distance between them is below the cut-off radius $r_{c}$. Beyond the cut-off radius $r_{c}$, there is no interaction between the particles. Moreover, ${\varvec{r}}_{{{\varvec{ij}}}}$ = ${\varvec{r}}_{{\varvec{i}}}$ − ${\varvec{r}}_{{\varvec{j}}}$, $r_{ij}$ =$\left| {{\varvec{r}}_{{{\varvec{ij}}}} } \right|$, and $\hat{\user2{r}}_{ij} = { }\left| {{\varvec{r}}_{{{\varvec{ij}}}} { }} \right|/{\varvec{r}}_{{{\varvec{ij}}}}$. Here, $a_{ij}$ denotes the maximum repulsion between the two interacting DPD beads ($i$ and $j$). The formula to calculate $a_{ij}$ was derived by Groot and Warren [292] which was obtained by matching the compressibility of the DPD fluid to that of water.

20 $$a_{ij} = \frac{{75k_{B} T}}{{\rho_{{{\text{DPD}}}} }}$$

where $k_{B} , T,$ and $\rho_{{{\text{DPD}}}}$ represent the Boltzmann constant, system’s equilibrium temperature, and density of the DPD system, respectively.

The dissipative force ${\varvec{F}}_{ij}^{D}$ is states as [293]:

21 $${\varvec{F}}_{ij}^{D} = - \gamma w^{D} \left( {r_{ij} } \right)\left( {\hat{\user2{r}}_{ij} \cdot {\varvec{v}}_{ij} } \right)\hat{\user2{r}}_{ij} ,$$

where ${\varvec{v}}_{ij}$ represents the relative velocity of the beads $i$ and $j$, and is defined as ${\varvec{v}}_{ij} = {\varvec{v}}_{i} - {\varvec{v}}_{j}$. The parameters $\gamma$ and $w^{D} \left( {r_{ij} } \right)$ are related to the dissipative force and are called dissipative force coefficient and dissipative force strength coefficient, respectively. As mentioned above, the dissipative force cools down the DPD system, because it tends to reduce the velocities of the interacting DPD beads, which is equivalent to removing part of the kinetic energy from the DPD system. Both the dissipative and random forces work in tandem to implement a thermostat. As a result, the temperature of the DPD system remains at a constant level with small fluctuations.

In principle, any integration technique that is utilized in MD simulations can be used in DPD simulations. However, since the velocity of DPD particles depends on the force, a modified version of the Velocity-Verlet integration technique [292] is utilized. The steps involved in the modified Velocity-Verlet technique are given below:

22 $${\varvec{r}}_{i} \left( {t + \Delta t} \right) = {\varvec{r}}_{i} \left( t \right) + \Delta t{\varvec{v}}_{i} \left( t \right) + \frac{1}{2}\Delta t^{2} \frac{{{\varvec{F}}_{i} \left( t \right)}}{{m_{i} }}$$

23 $$\tilde{\user2{v}}_{i} \left( {t + \Delta t} \right) = {\varvec{v}}_{i} \left( t \right) + \frac{1}{2}\Delta t\frac{{{\varvec{F}}_{i} \left( t \right)}}{{m_{i} }}$$

24 $${\varvec{F}}_{i} \left( {t + \Delta t} \right) = {\varvec{F}}_{i} \left( {{\varvec{r}}_{i} \left( {t + \Delta t} \right), \tilde{\user2{v}}_{i} \left( {t + \Delta t} \right) } \right)$$

25 $${\varvec{v}}_{i} \left( {t + \Delta t} \right) = {\varvec{v}}_{i} \left( t \right) + \frac{1}{2}\Delta t\frac{{{\varvec{F}}_{i} \left( t \right) + {\varvec{F}}_{i} \left( {t + \Delta t} \right)}}{{m_{i} }}$$

In the modified Velocity-Verlet scheme, since the force is velocity-dependent, the new velocity (denoted by $\tilde{\user2{v}}_{i}$) for the next time step $\left( {t + { }\Delta t} \right)$ is first predicted, and is later corrected in the last step. The force, however, is still updated in a single iteration, as is the case in the original Velocity-Verlet algorithm. As a result, the computational cost does not increase in the modified Velocity- Verlet technique.

## Molecular simulations of nanoparticles

### Lipid-based nanoparticles

As mentioned earlier, liposomes have the ability to encapsulate and transport drug molecules across selective cell membranes. Also, the morphology of liposomes is comparable to that of biological membranes. Furthermore, they have the ability to incorporate diverse compounds. All these features make liposomes an ideal candidate for drug delivery applications. Thus, liposomes have been thoroughly investigated over the past 50 years and they continue to be the topic of extensive research. To enhance the solubility and circulation times of liposomes, the surface of liposomes is often functionalized by coating them with a protective PEG layer in a process called PEGylation [294]. The PEG layer forms a steric sheath around the liposome surface that prevents the coating of liposomes by proteins, thereby decreasing the drug uptake by immune cells. Nevertheless, the manner in which PEGylated liposomes interact with other neighboring entities (such as solvent molecules and other lipids) to shape their localization and steric effect is still not clearly understood.

**Fig. 17:**
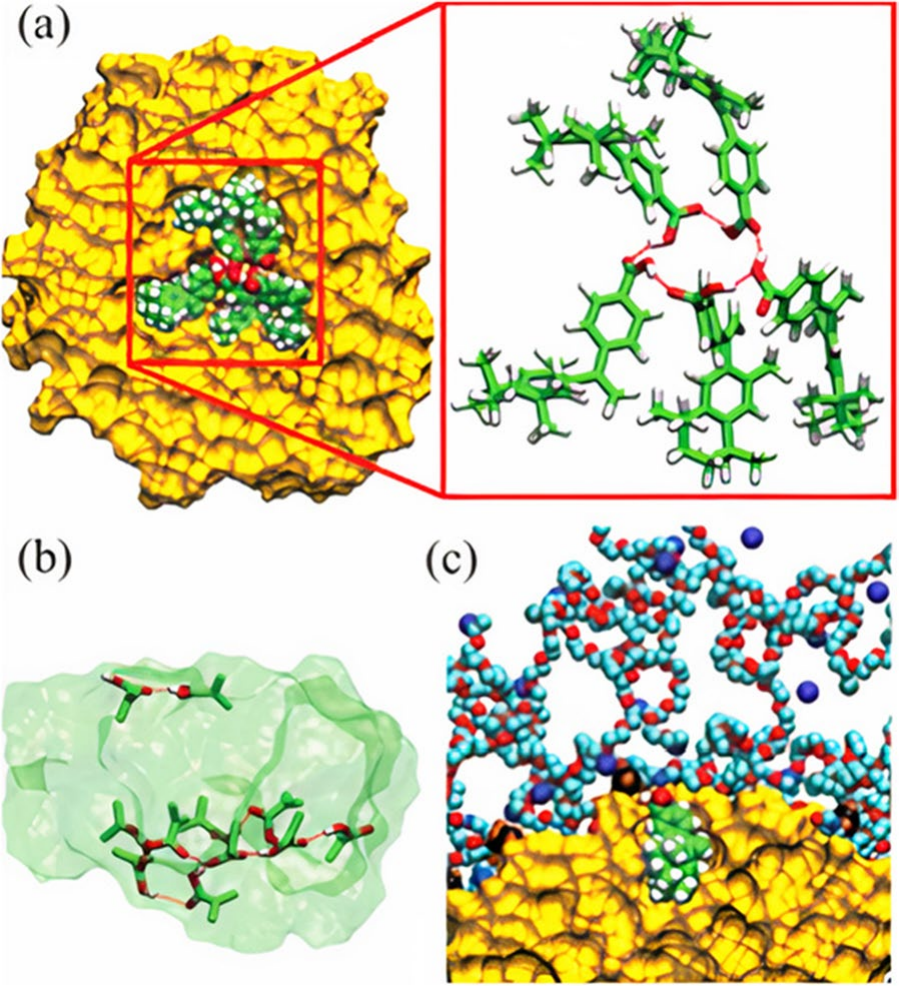
**a** The results of an AMD simulation showing the creation of a cluster of five bexarotene molecules inside the core of phospholipid-based sterically stabilized micelles core after 11 ns of equilibration. The zoomed-in figure details a chain of hydrogen bonds between the COOH groups. **b** Two networks of hydrogen bonds are formed in the cluster of 11 bexarotene molecules in the sterically stabilized micelles core After 17 ns of equilibration. **c** The nonpolar component of a solvated bexarotene molecule is directed toward the alkane core, while the COOH group is orientated toward the ionic interface. Reprinted from [297], Copyright 2013 American Chemical Society

Given the computational cost of doing AMD simulations of full liposomes, a typical strategy is to deduce conclusions about liposomes from simulations involving a lipid bilayer, which are less costly. Roccatano et al. [295] carried out atomistic molecular dynamics (AMD) simulations of PEGylated lipid bilayers, and the computations of their free-energy revealed significant interactions between PEG and the bilayer's lipid headgroups. Using AMD simulations, Dzieciuch et al. [296] investigated the impact of PEGylation on drug loading efficiency of liposomes. It was reported that PEGylation improved the drug-loading efficiency of membranes [296]. Vukovic et al. [297] utilized AMD simulations to investigate the binding mechanisms of two therapeutic agents, named bexarotene and human vasoactive intestinal peptide (VIP), in PEGylated phospholipid nanocarriers called sterically stabilized micelles (SSM). Their findings demonstrated that AMD simulations may be utilized to determine the solubility of drugs in nanocarriers. They showed that both Columbic and hydrophobic/hydrophilic interactions between the phospholipid polymers and drug molecules might contribute to the drug molecules' stabilization in the SSM. By refinement of these and other kinds of interactions between the drug and the nanocarrier, the drug could be attached to the targeted location of the nanocarrier (Fig. 17). Their work demonstrated that accurate atomistic simulations could provide critical insight into drug nanocarrier complexes, hence influencing the development of future nanomedicines.

**Fig. 18:**
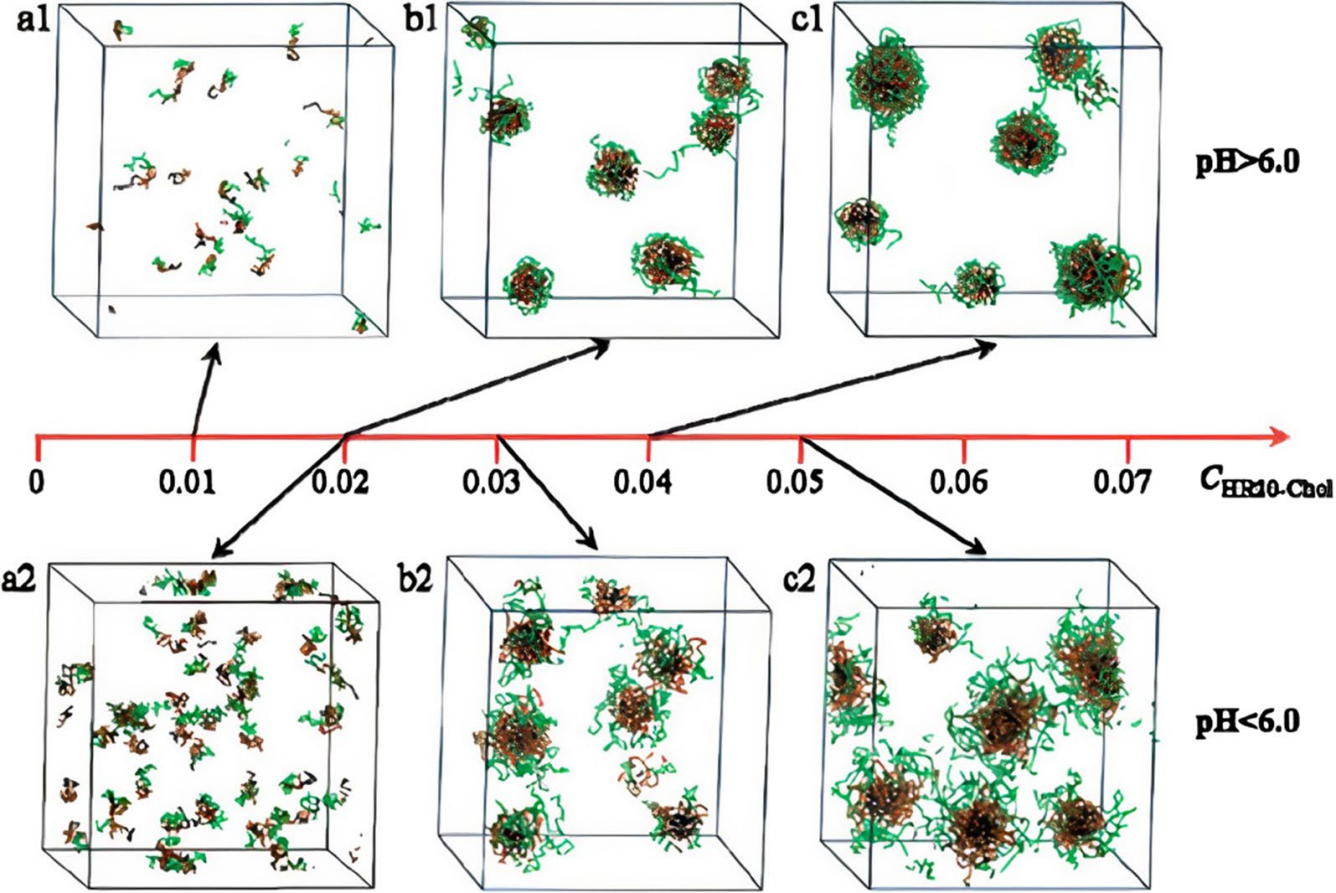
A Dissipative Particle Dynamics (DPD)-based investigation on the microstructures of micelles (HR20-Chol) at different pH levels of the aqueous solution. a1, b1, and c1 represent that cases where pH > 6, while pH < 6 for the cases shown in a2, b2, and c2. Line color legend: Green = Arginine; Brown = Histidine, and Black = Cholesterol. The water molecules are omitted in the figure for clarity. Reprinted with permission from ref. [303]. Copyright 2010 American Chemical Society

Another topic of particular interest in nanotechnology is the self-assembly of nanoparticles since it enables the development of novel nanoscale materials with desired qualities [298]. To this end, the CG MD and DPD simulations are better options for lipid self-assembly. By utilizing CG MD simulations, Janke et al. [299] investigated the phase behavior of oleic acid aggregation for a range of concentrations and protonation states. Based on the protonation state of the oleic acid head group, a variety of morphologies (such as vesicles, worm-like micelles, and oil phases) were formed. The analysis of lipid-based aggregates useful for drug administration has also been carried out using CG and DPD simulations [299–302]. For instance, Lee and Pastor [301] investigated the self-assembly of lipids and PEG-grafted (also called PEGylated) lipids in water with varying sizes and concentrations of PEGylated lipids using the Martini CG force field. Their simulations suggested that as the PEGylated lipid concentration was raised, the average aggregate size was reduced. Consequently, a self-assembly of liposomes, bicelles, or micelles are formed in the simulation depending on the concentrations of PEGylated lipid in the mixture. These numerical findings were consistent with the experimental data. Furthermore, PEGylated lipids were found to be more concentrated at the rims of bicelles. However, no such trend was observed in the lipids without PEG as their distribution was the same at the planar surfaces and rims. Guo et al. [303] employed the DPD method to investigate the microstructures of micelles at different pH levels. The micelles were prepared from the self-assemblies of cholesterol-conjugated peptides (HR20-Chol) and were either loaded with doxorubicin (DOX) or left blank. It was reported that when the pH is above 6.0, these micelles are quite dense and are better at loading DOX because of the hydrophobicity of histidine residues. This is different from the case when the pH is below 6.0 where the micelles start swelling. This change in structure is believed to make it easier for DOX to be released from the micelles' cores. The results were shown to be consistent with experimental findings (Fig. 18).

### Polymeric nanoparticles

Polymeric NPs are famous for their stability and ease of surface modification [304]. Like liposomes, these polymer-based nanoparticles have also found increasing applications in drug delivery systems (DDS). Amphiphilic polymers—composed of both hydrophilic and hydrophobic parts—may take on a wide range of structures in solvents. These include core–shell NPs and rod-like micelles as well as Janus particles and micelles. Numerous factors, such as temperature, solvent pH value and polarity, nature of components, and more that influence the self-assembly structures and stability, have been investigated by researchers. To accurately forecast the characteristics of polymers, it is critical to understand their chemistry and relate it to the required material qualities [305]. Quantum chemical calculations might help us better understand electronic characteristics, however in most cases, these calculations are restricted to oligomers and ignore conformational features or bulk morphology [306]. Molecular dynamics (MD) may be used to precisely model chemistry at the atomistic level. In an attempt to design a stable unimolecular star-block copolymer (SCP), Huynh et al. [307] conducted an AMD simulation of thirteen SCPs. Each SCP contained a central section and six connected arms, each having a hydrophilic (PEG) and hydrophobic (PCL) component. The SCPs had varying PCL and PEG lengths and molecular weights. A thick hydrophobic core surrounds a PEG shell in these core–shell structures. Partial water exposure of the PCL core leads to multi-molecular micelles, according to the simulations. Increasing PEG length protects the PCL core from water but increases micelle size. The lowest number of PEG units necessary to completely protect the PCL core is crucial since smaller micelles are favored in DDS applications. The quantity and molecular weight of PEG and PCL blocks were shown to be quantitatively related. Using DPD, Guo et al. [308] performed systematic simulations involving polymer blends. Their focus was to investigate how the hydrophobicity and compatibility difference between blended polymers and the solvent might affect the phase separation structures (core–shell or Janus) of a variety of polymer blends in solution. Chen and Ruckenstein [309] used DPD simulations to study the formation mechanism of a multicomponent multicore micelles (MMM) from two types of star-shaped copolymers. The degradation and dynamics of these MMMs were also reported, Taresco et al. [310] studied the structure and self-assembly of amphiphilic polyelectrolytes (APEs) in aqueous media using AMD simulations. Wang et al. [311] also used AMD simulation to characterize cholesterol functionalized CD micelles. With the addition of a second hydrophilic phosphatidylcholine group, Tan et al. [312] observed in their DPD simulations a phase shift from sphere-to-rod morphologies in multi-block polymer micelles.

**Fig. 19:**
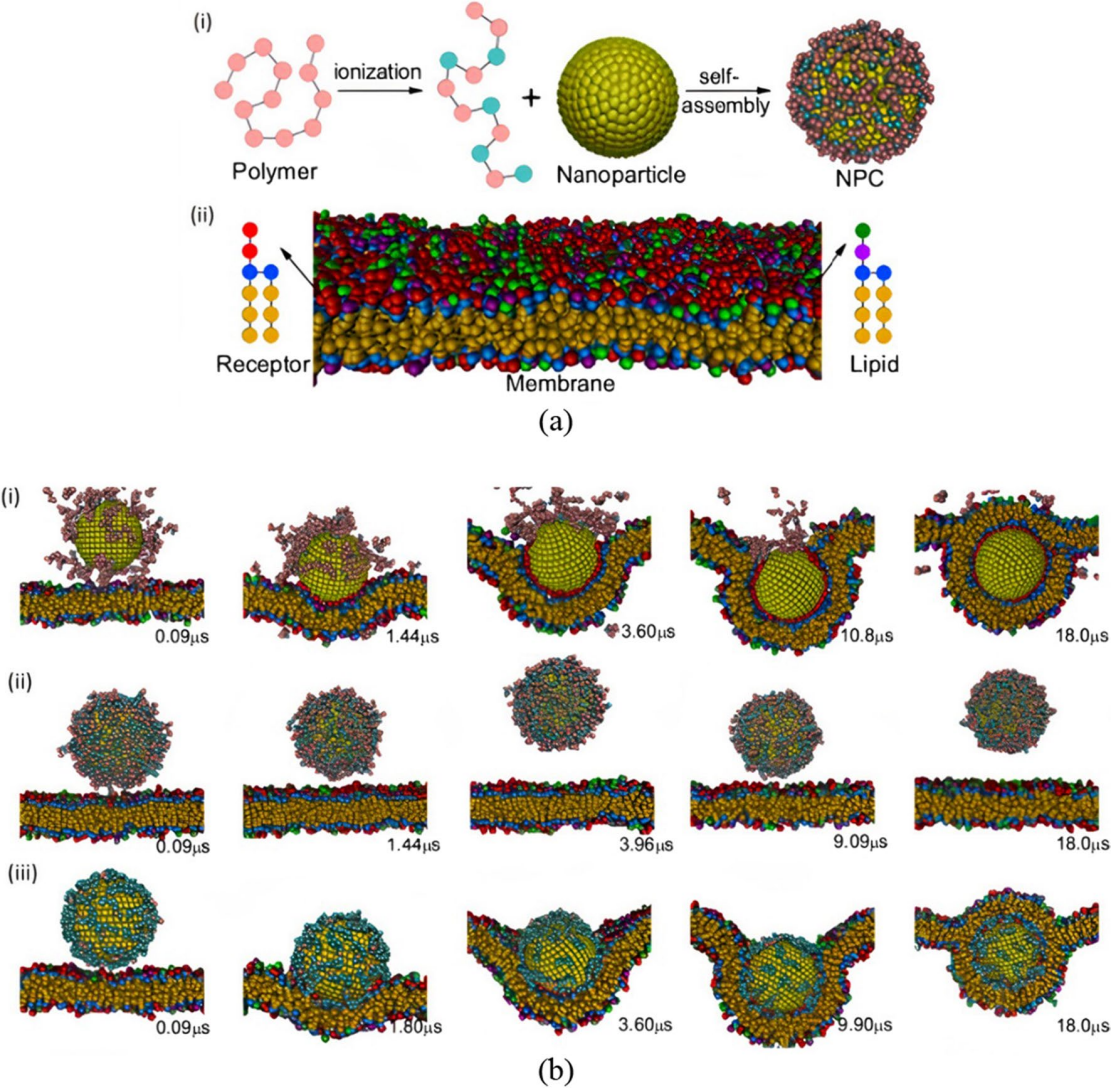
**a** The details of NPC (Nanoparticle-Polymer Complex). **a** (i) shows the assembly of pH-sensitive polymer on the nanoparticle surface. **a** (ii) represents the membrane details with lipids and receptors. Schematic illustration of the models in the simulations in which the nanoparticle-polymers complex is shown along with the membranes and architectures of receptors and lipids. **b** Time evolution of NPC endocytosis for three cases of pH. In **b** (i) and **b** (iii), the pH values are lower and higher than the polymer's pKa, respectively. Consequently, the membrane fully engulfs the NP. In contrast, in **b **(ii), the pH value is equal to polymer’s pKa. Therefore, the endocytosis is blocked. The green, purple, and blue beads correspond to the membrane lipid heads carrying a charge of + *e*, − *e*, neutral, respectively. Lipid tails and receptor heads are shown as orange and red beads, respectively. Moreover, the nanoparticles are represented by yellow beads and the polymer beads are depicted in cyan (carrying − *e* charge) and pink colored beads. Reprinted with permission from ref. [313] (Open Access)

Ding and Ma [313] used DPD to design pH-sensitive nanomaterials with the aim to (i) use the designed nanoparticle-polymer complexes in order to regulate nanoparticle cellular uptake for a variety of pH conditions, and (ii) comprehending the receptor-mediated endocytosis mechanism of pH-sensitive nanoparticle-polymer complexes. In their model, each lipid comprised a headgroup with four linked hydrophilic beads (H) and two tails with three hydrophobic beads (T) each. The first head bead was charged + e, while the second was charged − e; the remaining two beads were uncharged. When modeling negatively charged lipids, a neutral hydrophilic bead replaces the first positive charged bead in the lipid molecule. The receptor molecule's first two head beads (R) were uncharged and could interact with the ligand bead (L) through mild L–J potentials. Through their 18-µs-long simulations under 03 three different pH values, they demonstrated that the endocytosis process was triply-pH-responsive. That meant that nanoparticles could only be engulfed by cell membranes at high and low pH values. The endocytosis process was blocked when the pH values were in the middle range (Fig. 19).

### Metallic-nanoparticles

**Fig. 20:**
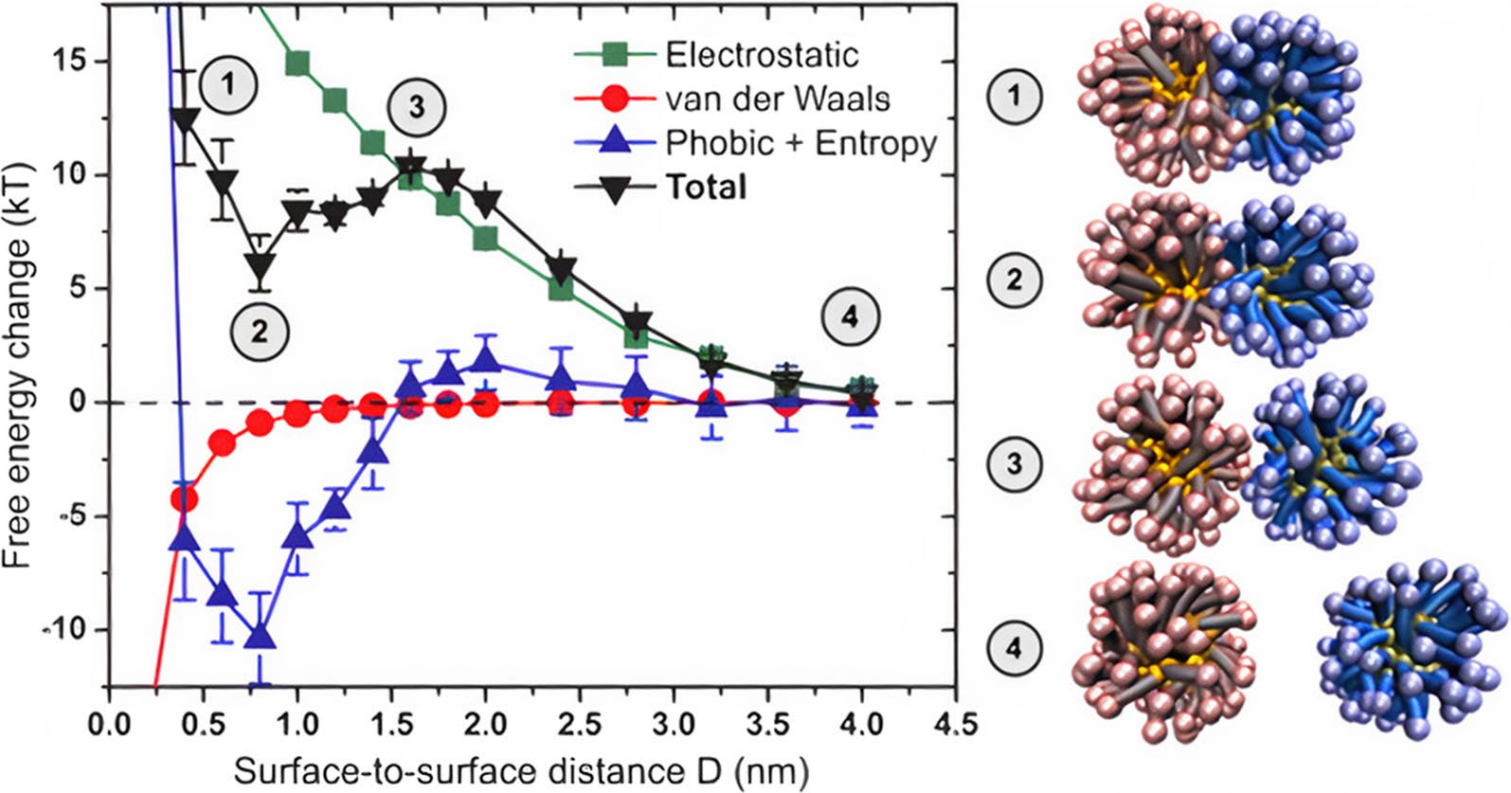
Graph showing change in the free energy components as a function of the surface-to-surface separation distance between two AuNPs. The corresponding states of the system at different zones are also shown for four cases [322]

Among metallic nanoparticles, gold nanoparticles (AuNPs) are of special interest owing to their innumerable positive attributes. AuNPs are biocompatible, stable, easy to synthesize, and can be manufactured in a broad size range. Although the biocompatibility of AuNPs is comparatively less than that of liposomes and other biomolecules, their widespread applications in medicine and biology make them extremely useful. Some of the major applications of AuNPs include biosensing, imaging of tumor cells, cancer irradiation therapy, probing endocytosis, and the development of targeted drug delivery systems [314–316]. The dimensions of AuNPs range from a few nanometers to a few hundred nanometers, and their different shapes include cubes, spheres, prisms, tetrahedrals, rods, and more [317, 318]. An AuNP is typically comprised of a hard Au core that is inert in nature and is meant to provide stability to the particle structure. The core is coated/functionalized with a monolayer of ligands that are used to tune the surface properties (e.g., hydrophobicity, charge) of the AuNP. The ligands can also be loaded with numerous small molecules such as proteins, peptides, and DNAs [319]. Numerous computational investigations into AuNP characteristics and their interactions with other molecules have been conducted by the researchers. Lee and Ytreberg [320] used MD simulations to investigate the structure and dynamics of six peptides and the impact of conjugating them to a gold nanoparticle. The peptides were present in water either unbounded or conjugated to AuNPs. The researchers reported that conjugation affected both the peptide structure and its dynamics. Peptides that lacked secondary structure tended to adsorb to the surface of AuNPs at various locations along the peptide, possibly restricting their ability to interact specifically with cell media. This makes the peptides having secondary structures in the solution favorable candidates for drug delivery as they promote peptide-NP conjugation. Brancolini et al. [321] performed numerical simulations at different length scales (*ab-initio* QM and AMD) to investigate the interactions between AuNPs and a cysteine-free protein called Ubiquitin. Van Lehn et al. [322] conducted numerous implicit-solvent numerical simulations on lipid-coated AuNPs to understand the underlying mechanism of undesired AuNPs aggregation in the monolayer-protected AuNPs for applications like drug delivery systems (Fig. 20). This understanding is crucial as it can lead to the synthesis of optimized protecting monolayers that can suppress aggregation or dispersion based on the application. Their simulations comprised two AuNPs initially separated by a distance $D = \infty$ and were brought closer to the desired distance. The change in free energy was computed with the decrease in the surface-to-surface distance of the AuNPs. The diameter of AuNPs was between 1.0 and 3.0 nm, each shielded by a monolayer of alkanethiol ligand, and was assumed to be charged. It was shown that the aggregation was prompted by ligand deformation and was a function of AuNP size, the length and type of ligands used, and other environmental conditions [322].

## Conclusions, outlook and future aspects

### System complexity versus process control

Synthesizing NPs using active microfluidics offers better process control at the expense of complex system configuration, where additional modules such as a signal generator, a piezoelectric actuator, etc. are needed to actively control mixing inside microchannels. However, passive microfluidic systems generally require a microchannel connected to one or more pumping units, where the operational flexibility is limited to flow rate modulations only. A trade-off between the complexity of the microfluidic systems and their flexibility in operation enable a wide range of platforms for NPs synthesis depending upon the available instruments and resources, and the desired NPs characteristics and throughput. For example, SAW-based acoustic streaming flow provides efficient spatio-temporal control of the mixing phenomenon inside the microchannels, however, a multi-steps fabrication process is needed to deposit metallic IDTs on a piezoelectric (LiNbO_3_) substrate and attach a PDMS microchannel on top. Oxygen plasma binding of LiNbO_3_ and PDMS is a non-trivial process, which requires deposition of an additional SiO_2_ layer on top of the substrate. On the other hand, single layered passive micromixers, which are fabricated using relatively simpler microfabrication processes, offer straightforward mixing mechanisms for NPs synthesis, however, with limited control of the reagents mixing process. The mixing efficiency of passive microfluidic platforms can be enhanced by using complex microchannel geometries, e.g., split and recombine designs with multi-layered 3D devices, however, the fabrication complexity increases for such sophisticated microchannel designs. The passive micromixers, with their simple operation and a range of possible designs, are relatively more prone to clogging and fouling in comparison to the active approaches. Overcoming the corresponding limitations of the active and passive methodologies and marrying their advantages in hybrid microfluidics platforms (combination of active and passive mixing) are subjects of the active research in this area. For example, by utilizing coaxial glass capillaries to generate a co-axial flow of reagents, Othman et al. [140] synthesized polymer NPs where the organic phase was completely surrounded by the aqueous phase with no direct contact with the outer walls. This increased the contact surface area and prevented polymer precipitation/fouling on the channel walls. Fouling in the microfluidic channels can also be prevented by acoustic actuation within channel or sonication of the whole device. For example, Ozcelik and Aslan [117] used piezoelectric transducers glued to a rectangular glass capillaries to vigorously mix precursors for NPs production and prevent channel fouling due to the high shear acoustic streaming flows.

### Challenges

The integration of microfluidic technology with NP synthesis has solved several challenges associated with conventional/bulk NP production, quality control and reproducibility [323]. However, difficulty to produce NP in quantities (in grams/h) suitable for intended applications still impedes the translation of microfluidic NPs from laboratory to large-scale commercial production. Several ideas have been proposed to address this obstacle which includes parallelizing the production process, increasing the throughput per microchannel by utilizing novel device designs, etc. Production parallelization offers scale independency and can be achieved by fabricating identical channels simultaneously on the same platform to ensure identical operating conditions and physicochemical properties across the channels. An appropriate microchannel design could help in improving the throughput and quality of the produced NPs. For example, Hood and DeVoe [324] used a COC microchannel with a high aspect ratio of 100:1 to produce liposomes with reasonable size and PDI at a mass flow rate of 100 mg/h, compared to 3 mg/h possible with a standard microchannel design (aspect ratio 0.5:1). Similarly, Pourabed et al. [218] used a purposefully built PDMS-silicon-PDMS microchannel with a lotus shaped cavity to acoustically mix precursors at high flowrate of 1.2 mL/min within a short time scale (2 ms) and produce 52 nm PLGA NPs. These afore mentioned challenges could be overcome with continuously improving device designs and experimental procedures. However, another challenge that also needs to be addressed is to encourage and convince the pharmaceutical industry to adopt and promote microfluidic platforms for high throughput and high quality production of NPs.

### Future direction of research

A long term goal of integrating microfluidics with NP/drug synthesis is the ability to customize therapeutics for individual or a subset of patients, and overcome challenges related to population heterogeneity that reduce the overall efficacy of medicines [15]. This personalized therapeutic tailoring approach is known as “Precision Medicine”, and it utilizes information such as genetic profile, and patients’ history to implement a personalized treatment plan with specifically synthesized therapeutics. One approach to accomplish such an ambitious project is ‘Pharmacy-on-Demand’—a strategy to incorporate the entire drug manufacturing process in a single platform at a hospital or a pharmacy [325]. To realize this vision, multiple high throughput micromixers and flow reactors can be used with other analytical systems for the synthesis, characterization, evaluation and quality assessment of the NP-based therapeutics.

For the near future research, there is a growing interest within the microfluidics field in exploring alternative materials and fabrication methods for the micromixers. Conventional microchannel fabrication methods include soft lithography (PDMS), and dry/wet etching (silicon). These methods result in channels with high resolution and quality; however, specialized cleanroom facilities and expert users are required to conduct these processes, potentially hindering their widespread adoption within the research community. Therefore, alternative low-cost materials are being explored that can be easily processed without a cleanroom facility, and can transition to the commercial market economically. Alternative materials include polymers such as cyclic olefin copolymer (COC) and poly(methyl methacrylate) (PMMA). These thermoplastics are transparent and versatile, and can be used to fabricate microchannels via hot embossing, injection molding, micromachining, laser ablation, and 3D printing [83]. For example, Aranguren et al. [156] fabricated PMMA serpentine microchannels via laser ablation, and demonstrated production of lipid NPs (188 nm) at a maximum flow rate of 5 mL/min. Similarly, Liu et al. [174] fabricated a PMMA double-layer Y-shaped split and recombination micromixer by computer numerically controlled micromachining for AgNPs synthesis. Additionally, COC has been utilized in commercially available microfluidic platforms such as, Dolomite Microfluidics (Royston, UK) [326], and the NanoAssemblr™ benchtop, which have been used extensively in liposome synthesis and research [88, 158–163]. Microfluidic devices fabricated in disposable COCs can also be recycled by melting at high temperatures to ensure sterilization [327].

Another research direction that is gaining momentum is the hybrid approach. Where one approach is concerned with the combination of passive and active mixing techniques in a single platform (hybrid mixing), and another related to organic-organic, organic–inorganic, and inorganic-inorganic hybrid NPs. For instance, hybrid micromixing was demonstrated by Bachman et al. [241] where acoustically actuated sharp edges (active mixing) and tesla structures (passive mixing) were implemented to enable a wide range of flow rates (20–2000 µL/min) for production of smaller PLGA NPs (64.5–93.76 nm). Moreover, hybrid NPs composed of polymer (core)-lipid (shell) bilayers provide the mechanical properties of biocompatible polymer NPs with the biomimetic advantages of lipid NPs [328], such work was demonstrated by Liu et al. [209], synthesizing PLGA NPs coated with exosomes. Nanoparticle functionalization is another method of engineering NP surfaces with groups such as monomeric stabilizers (e.g., thiol group), inorganic materials (e.g., gold or silica), and organic polymers (e.g., polyethylene glycol), optimized based on specific biomedical applications [329]. Where the purpose of NP functionalization is to mimic cellular surface compatibility to enable and aid in applications such as drug delivery systems, diagnostics, and drug discovery. Integration of functional groups (multifunctionalization) allows for multimodality therapeutic strategies or overcoming all limitations of the base NP. For example, Zhu et al. [330] developed a multifunctional NP (dendrimer-entrapped AuNPs covalently linked with α-tocopheryl succinate) that can be used for targeted cancer imaging and therapy.

Moreover, artificial intelligence and machine learning have been employed to assist and predict experimental characteristics in microfluidics synthesized NPs [331]. Where machine learning techniques are capable of building complex mapping relationships between existing data to determine the most effective combination of experimental conditions including temperature, concentration of precursors, and flow rates [332]. This optimizes the formulation process and makes it more energy efficient and cost effective, and can aid in the discovery of new drug candidates. For example, AgNPs with a specific absorbance spectrum was predicted by a deep neural network which built a link between the chemical composition and optical properties [333].

In conclusion, conventional NP production synthesizes NPs with a wide size distribution and batch-to-batch variation in physicochemical properties along with the requirement of additional chemical and physical processes. Microfluidics has the ability to overcome these limitations by precisely controlling the fluid flow, NP size, size distribution, and encapsulation efficiency. Here we have presented an overview of the fundamental mechanisms of active and passive mixing in microfluidics to produce NPs for potential use in biomedical applications and outlined the different classes of NPs. In addition, we have highlighted the advantageous reasoning behind the utilization of microfluidics and presented a detailed review of the recent advances in organic and inorganic NP synthesis. However, despite the recent progress and the rapidly evolving microfluidic field, more focus is needed on the overall process of NP production, from the initial phase of NP structure and composition consideration to engineering and manufacturing considerations such as device fabrication techniques and scalable production. Furthermore, the role of molecular-based numerical simulations in nanoparticle synthesis applications in numerous interesting fields, such as drug delivery, were discussed. Finally, an outlook on the complexity, challenges, and future direction of microfluidic NP synthesis was discussed.
